# A Review of Electromagnetic Induction on Planetary Bodies

**DOI:** 10.1007/s10712-025-09896-6

**Published:** 2025-08-04

**Authors:** Anna Mittelholz

**Affiliations:** Department of Earth and Planetary Sciences, ETH Zurich, Sonneggstr. 5, 8092 Zurich, Switzerland

**Keywords:** EM sounding, Planetary, Induction, Geomagnetism, Magnetosphere, Planetary geophysics

## Abstract

Electromagnetic (EM) sounding of planetary bodies other than the Earth was first possible on the Moon, but has since been used to probe interior structure of planets and moons throughout the solar system. This emergence has been facilitated by the growing availability of mission data and associated improved understanding of planetary magnetic field environments. In this review, I outline the general principles of EM induction, with particular emphasis on planetary specific assumptions and aspects that have to be considered in non-terrestrial environments, including limited or incomplete datasets. I review magnetic field mission data from past and ongoing space missions that can support EM investigations. The availability and quality of such data determine the scope and depth of investigations, ranging from characterizing most interior layers to identifying subsurface oceans. Looking ahead, upcoming missions will facilitate a better characterization of planetary bodies, which will contribute to addressing most fundamental questions, including the possibility of oceans and potential for life within the Jovian system.

## Introduction

Electromagnetic (EM) sounding has a long-standing history of allowing us to look into the interior of a planetary body. By exploiting time-varying magnetic fields, ubiquitous throughout our solar system, it represents a method that can be universally applied. EM sounding studies have naturally been dominated by studies focused on Earth, and multiple reviews summarize the underlying principles and continuous advances in the field (e.g., Rikitake 1973; Olsen 1999; Kuvshinov 2012; Grayver 2024). Less focus has been directed toward sounding of extraterrestrial bodies and only few authors provided overviews in this field: The early active extraterrestrial exploration efforts were directed toward the Moon, and reviews thus summarize the outcomes of these missions including their insights on lunar electrical conductivity structure and the surrounding magnetic field environment (Dyal et al. 1974; Vanyan 1980; Sonett 1982). Some later reviews included a planetary focus, highlighting applications of EM induction studies for other planets (Olsen 1999; Grayver 2024); one review focuses on planetary EM, but was written more than 10 years ago (Saur et al. 2010), and much development in the field has occurred since. The status of the literature in planetary sciences relies heavily on past, ongoing, and planned missions, and recent active space exploration efforts warrant an updated summary of EM studies focused on planetary bodies other than Earth.

**Fig. 1:**
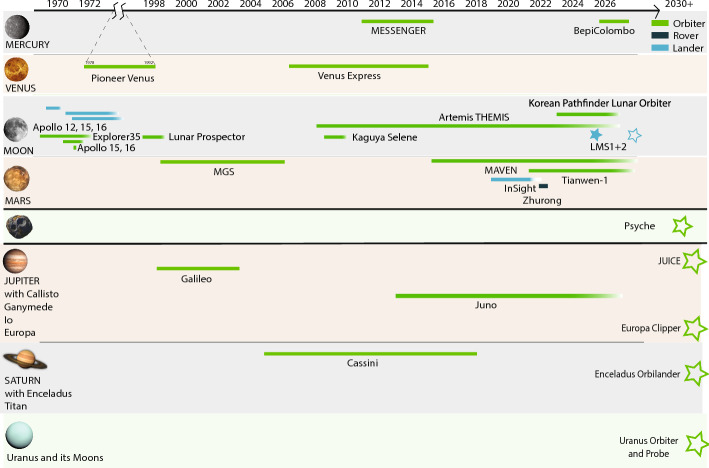
A timeline for planetary missions carrying a magnetometer in orbit or at the surface (flyby’s are not listed). Note the gap from 1974–1996, in which only one magnetometry mission, Pioneer Orbiter on Venus operated. See Table 1 for more info on individual missions. Hollow stars indicate upcoming missions

Additional and distinct challenges arise when exploring extraterrestrial bodies compared to the Earth, often due to (1) complex magnetic environments and (2) data limitations. First, magnetic environments around solar system bodies are unique. Those environments in combination with dynamic characteristics of the planet (e.g., orbital rotation) determine the geometry and frequencies of the inducing field. They determine whether a potential field description is possible or if an inducing field can be reduced to a simple field geometry, such as those driven by the mean equatorial ring current (Banks 1969). Thus, it is important to reconsider assumptions made commonly for the Earth, as they might not hold for other planetary bodies. Second, progress in planetary sciences is strongly dependent on planetary missions and the availability of data or the lack thereof. The acquisition of extraterrestrial data is timely and costly, and severe data limitations are common. However, despite this challenge, Fig. 1 shows that in the last 10+ years many missions have been actively measuring magnetic fields across the solar system. While planetary data might be sparse compared with terrestrial data, they do exist and are constantly growing. Most magnetic field data come from missions exploring the inner solar system: First, Mercury has been characterized in great detail by MESSENGER and is currently awaiting the arrival of BepiColombo. Venus was visited by two spacecraft. Further, the decade of the Moon has been announced as many nations and space agencies have once again started to land on the Moon, to provide a pathway for science on the Moon and beyond. Lastly, Mars data sets have expanded greatly in recent years, in form of orbiters, landers, and rovers. For the gas giants and their moons, past and recent data sets have enabled investigations, some summarized in earlier reviews (Saur et al. 2010). Findings of those earlier missions, particularly those of ocean worlds in the outer Solar System, have sparked interest, and particularly the Jovian system is an upcoming destination for two missions currently en route—JUICE and Europa Clipper. This development and ongoing and planned exploration efforts have influenced the research directions of the community.

Another difference from terrestrial studies is the kind of questions that are usually posed. In general, and because geophysical data is often sparse, in some cases most fundamental questions still exist. Unknowns range from large-scale structure and composition of planetary bodies, e.g., the presence of a core, to more specific questions such as the presence of an ocean or interior abundance of volatiles. Generally, the abundance of volatiles in our solar system is of great importance. In the case of Mars or Venus, it is unclear what happened to the water that once existed; did it escape to space or is it captured in the crust? What plays a role in the emergence of plate tectonics? These aspects are not meant to represent the range of unknowns, but they illustrate how fundamental the outcome of EM studies can be in addressing broader questions of planetary formation, evolution, and habitability.

EM sounding is based on time-varying inducing magnetic fields generating currents and secondary induced magnetic fields. The nature of this interaction is determined by the intrinsic property, electrical conductivity, which in turn is influenced by volatile content and mineralogy of the interior. There are different approaches to derive electrical conductivity from data, but all require separating inducing and induced fields. Generally, more than one magnetometer is required for a separation of those contributions. This additional information may come from a known dominant spatial structure of the inducing field, such as the terrestrial equatorial ring current (Banks 1969). It can also be obtained through measuring the electric field, as in magnetotelluric studies, or through simultaneous  magnetic field observations from multiple spacecraft. Because no combined electric and magnetic field measurements have enabled MT studies so far, the focus for inner solar system bodies is on (a) understanding and approximating the inducing field, i.e., the external magnetic field environment, or (b) the use of an additional spacecraft which measures the inducing field concurrently (Grimm and Delory 2012). For outer solar system bodies, the sparsity of data typically limits EM studies to testing specific hypotheses, such as the existence of a highly conductive layer, as expected for a subsurface ocean.

**Fig. 2:**
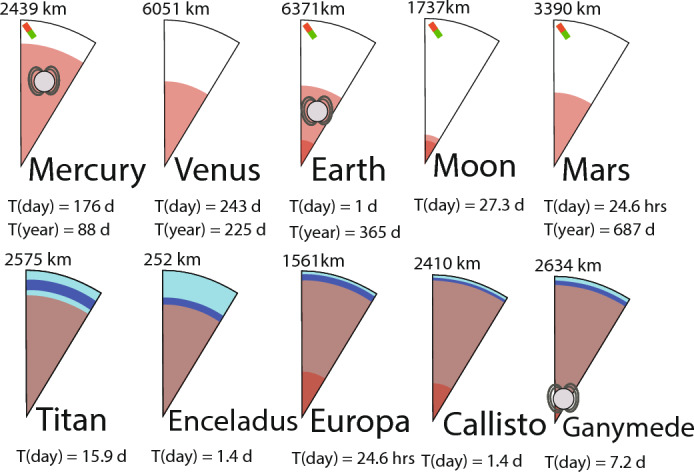
(Upper) Terrestrial planets and their approximate core-mantle boundaries (see references in respective sections below) and (Lower) so-called “ocean worlds”, Jupiter and Saturn moons that have been hypothesized to host oceans. The proposed transition from ocean (dark blue) / ice (light blue) to mantle and mantle to core are shown (Marusiak et al. 2021). Note however that the actual interfaces are not always well constrained. The respective planetary radii, durations for a full day, and—where applicable—the length of a year in units of Earth days are shown. The dynamo symbol indicates a current day dynamo, magnets indicate that crustal magnetization has been detected

Generally, the penetration depth of electromagnetic waves depends on the period, T, and the material they propagate through. This depth can be approximated by the skin depth, $d=\sqrt{\dfrac{\rho _a T}{\mu _o\pi }}$, where the apparent resistivity, $\rho _a$, is the resistivity of a half-space equivalent to the depth-dependent subsurface material through which the wave has propagated, and $\mu _0$ is the magnetic permeabilty of free space. Thus, longer periods can sound greater depths, and the frequency content of the inducing field determines which depths can be investigated. For any given period infinite models of internal structure can explain observations. However, with observations at multiple periods, one can constrain the interior structure. For many planets, the diurnal or annual period and their harmonics, e.g., the inducing field associated with planetary rotation/orbital motion, represent a recurring pattern of an inducing field. However, in cases like Venus where one day or year lasts several hundred Earth days, such periods can be less useful. High resistivity (or low conductivity) regions promote deeper penetration. Highly conductive regions are planetary cores due to their high iron content, or oceans that are expected for some of the Moons in the outer Solar System. The crust of terrestrial bodies can be very resistive, especially for bodies with low volatile and/or iron content, like the Moon. Figure 2 lists the size, layering, and orbital characteristics of important terrestrial bodies and ocean worlds in our Solar system.

EM sounding has been well developed and extensively applied on Earth, and I refer to a recent comprehensive review of magnetic sounding for more detailed background and methods (Grayver 2024). As such, in this review I will provide an overview meant to aid in connecting the basics of terrestrial literature with concepts that have been used in the planetary literature. I will focus on aspects that differ from terrestrial studies, particularly available data sets and their characteristics, as well as the magnetic environments of bodies in our Solar System. I will then review existing studies that have used EM sounding to explore the interior of planetary bodies and provide a summary that can be built on in the future.

## Methods

### Basic Equations

Maxwell’s equations describe the spatio-temporal behavior of electric and magnetic fields and the basic principles of EM induction. Specifically, Ampere’s law describes the generation of magnetic fields from electric currents as

1 $$\begin{aligned} \nabla \times \vec {B} = \mu _0 \vec {j}, \end{aligned}$$

and Faraday’s law describes the generation of electric currents by time-varying magnetic fields as

2 $$\begin{aligned} \nabla \times \vec {E} = -\dfrac{\partial \vec {B}}{\partial t}. \end{aligned}$$

$\vec {B}$ is the magnetic field, $\vec {j}$ the electric current density, $\vec {E}$ the electric field, and $\mu$ the magnetic permeability with $\mu _0 = 4\pi \times 10^{-7} \mathrm {Vs/Am}$, i.e., its value in free space.

The current $\vec {j}$ can be represented by

3 $$\begin{aligned} \vec {j} = \sigma \vec {E} + \dfrac{\partial \vec {D}}{\partial t} + \vec {j_\textrm{ext}}, \end{aligned}$$

where $\sigma$ is electrical conductivity, the second term represents the so-called displacement currents, and the last term represents the primary current. For terrestrial EM studies (and typical time scales of $10^{-3}$ s and longer), the second term is usually neglected because $\sigma \mu _{0} c^2>> \omega$, known as the quasi-static assumption, where *c* is the speed of light and $\omega$ is the angular frequency. This assumption implies that the magnetic field diffuses and does not propagate into the conductive material.

Thus, and from Eqs. 2 and 1 and the isotropic Ohm’s law (Eq. 3), the induction process within a body or conductive fluid at rest is described by

4 $$\begin{aligned} \dfrac{\partial \vec {B}}{\partial t}= -\nabla \times \left( \dfrac{1}{\sigma \mu _0} \nabla \times \vec {B}\right) , \end{aligned}$$

or for constant conductivity,

5 $$\begin{aligned} \dfrac{\partial \vec {B}}{\partial t}=\dfrac{1}{\sigma \mu _0}-\nabla ^2 \vec {B}. \end{aligned}$$

Due to the linearity of the induction equation, time-domain fields can be represented as a superposition of harmonic (Fourier) components. This makes it convenient to work in the frequency domain, where each frequency component of the inducing field produces a corresponding response. Specifically, we assume harmonic time dependence of the form

6 $$\begin{aligned} \vec {B}({\textbf {x}}, t) = \tilde{\vec {B}}({\textbf {x}})e^{i\omega t}. \end{aligned}$$

where $\tilde{\vec {B}}$ is the complex amplitude of the field and $k=\sqrt{i\omega \mu _0\sigma }$ $\omega$ as the angular frequency of the inducing field oscillations. This allows the diffusion equation to be expressed as the Helmholtz equation inside the conductor and Laplace’s equation outside.

Within the conducting body, the magnetic field obeys the Helmholtz equation, where the complex wavenumber is defined as $k=\sqrt{i\omega \mu _0\sigma }$.

7 $$\begin{aligned} \nabla ^2\vec {B} = -k^2 \vec {B}, \end{aligned}$$

Outside the conductive body and in a current-free region the solution is governed by Laplace’s equation and

8 $$\begin{aligned} \nabla ^2\vec {B} = 0. \end{aligned}$$

### Potential Representation

If the region between a planetary surface and external field sources can be approximated as source-free, Eq. 1 reduces to $\nabla \times \vec {B} =0$. Thus, $\vec {B}$ is a potential field and $\vec {B} = -\nabla V$, where *V* is a scalar potential. Because $\vec {B}$ is solenoidal, a scalar potential, *V*, satisfies Laplace’s equation

9 $$\begin{aligned} \nabla ^2 V = 0, \end{aligned}$$

and can be represented by the sum of its external and internal contributions (Backus et al. 1996),

10 $$\begin{aligned} \begin{aligned} V(\vec {r}, \omega )&= V_\textrm{ext}(\vec {r},\omega ) + V_\textrm{int}(\vec {r},\omega ; \sigma ) \\&= a \sum _{n=1}^{N} \sum _{m=-n}^n \left( \epsilon _n^m (\omega ) \left( \frac{r}{a} \right) ^{n} + \iota _n^m (\omega ) \left( \frac{a}{r} \right) ^{n+1} \right) P_n^m(\cos \theta ) e^{im\phi }\\ \end{aligned} \end{aligned}$$

where *a* is the reference planetary radius, $P_n^m$ are the associated Schmidt semi-normalized Legendre functions, *N* is the maximum degree of external and internal expansion (Gauss) coefficients $\epsilon _n^m$ and $\iota _n^m$. From Eqs. 9 and 10, the magnetic field components can be expressed as:

11 $$\begin{aligned} B_r(\vec {r},\omega )= & -\sum _{n,m} \left[ n \epsilon _n^m (\omega ) \left( \frac{r}{a}\right) ^{n-1} - (n+1) \iota _n^m (\omega ) \left( \frac{a}{r}\right) ^{n+2} \right] P_n^m(\cos \theta ) e^{im\phi } \end{aligned}$$

12 $$\begin{aligned} B_{\theta }(\vec {r},\omega )= & -\sum _{n,m} \left[ \epsilon _n^m (\omega ) \left( \frac{r}{a}\right) ^{n-1} + \iota _n^m (\omega ) \left( \frac{a}{r}\right) ^{n+2} \right] \dfrac{dP_n^m(\cos \theta )}{\partial \theta } e^{im\phi } \end{aligned}$$

13 $$\begin{aligned} B_{\phi }(\vec {r},\omega )= & -\sum _{n,m} \left[ \epsilon _n^m (\omega ) \left( \frac{r}{a}\right) ^{n-1} + \iota _n^m (\omega ) \left( \frac{a}{r}\right) ^{n+2} \right] \dfrac{im}{\sin \theta } P_n^m(\cos \theta ) e^{im\phi } \end{aligned}$$

Note that although the scalar potential can also be expanded in the time domain, the frequency-domain formulation is convenient here because of the linearity of induction and the frequency dependence of the response. Either way Gauss coefficients can be estimated from a time series of magnetic field measurements. For terrestrial studies, the dominant structure is described by $n,m=1,0$ due to the ring current, but other sources can also be exploited (Grayver 2024).

### Useful Analytical Solutions

Due to often extremely limited data, it can be useful to seek analytic solutions for a sphere (Parkinson 1983) that can approximate observed or hypothesized geometries.

#### Spherical Shell Geometry

**Fig. 3:**
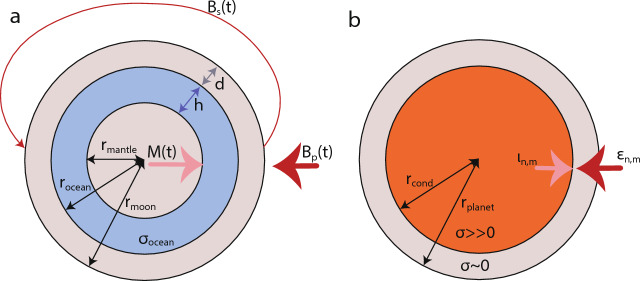
**a** Geometry of the three-layer shell model as introduced by Zimmer et al. (2000), commonly used to test the ocean hypothesis. $B_p$ is the primary inducing field and $B_s$ is the secondary field due to the magnetic dipole moment *M*. Ocean thickness is denoted by *h*, ice thickness by *d*. **b** The spherical shell model with a highly conducting interior of radius $r_\textrm{cond}$ overlain by an insulating mantle layer and a total radius of $r_{planet}$ as proposed for Mercury (Grosser et al. 2004). Inducing and induced fields of spherical harmonic degree and order *n* and *m* are denoted by $\epsilon$ and $\iota$

A suitable approach to modeling the response of a possible subsurface ocean is to use a spherical shell model (Fig. 3a), where a conductive ocean is bounded by an insulating mantle and surface material (Zimmer et al. 2000; Saur et al. 2010). For such a model, an analytical solution exists (Srivastava 1966; Parkinson 1983).

An oscillating primary field, $\vec {B_p}$, in the direction of the unit vector $\hat{e}$ with angular frequency $\omega$ can be written as

14 $$\begin{aligned} \vec {B_p} = B_p e^{-i \omega t} \hat{e}. \end{aligned}$$

If the primary field is uniform and $\sigma$ spherically symmetric, the induction response outside the conductor, i.e., $r>r_\textrm{ocean}$, is a dipole field (Parkinson 1983),

15 $$\begin{aligned} \vec {B_s} = \dfrac{\mu _0}{4 \pi } [3(\vec {r} \cdot \vec {M}) \vec {r} -r^2\vec {M}] / r^5. \end{aligned}$$

with its moment, $\vec {M}$, oscillating at the same frequency and along the same direction as the primary field and with amplitude *A*,

16 $$\begin{aligned} \vec {M} = -\dfrac{2 \pi }{\mu _0} A e^{i \phi } \vec {B_p} r_\textrm{moon}^3. \end{aligned}$$

For the spherical shell geometry, Eq. 5 can be solved where the radial component of the solution is given by Bessel’s equation of the first kind, under the assumption of suitable boundary conditions including continuity across the spherical shell boundaries (Zimmer et al. 2000). As such, the amplitude of the induction response is $A \epsilon [0,1]$, and $\phi \epsilon [0,2 \pi ]$ represents the phase lag between the primary inducing and the secondary induced field and

17 $$\begin{aligned} A e^{i \phi }=\left( \frac{r_{\text{ ocean } }}{r_{\text{ moon } }}\right) ^3 \frac{\xi J_{5 / 2}\left( r_{\text{ ocean } } k\right) -J_{-5 / 2}\left( r_{\text{ ocean } } k\right) }{\xi J_{1 / 2}\left( r_{\text{ ocean } } k\right) -J_{-1 / 2}\left( r_{\text{ ocean } } k\right) }, \end{aligned}$$

where

18 $$\begin{aligned} \xi =\frac{r_{\text{ mantle } } k J_{-5 / 2}\left( r_{\text{ mantle } } k\right) }{3 J_{3 / 2}\left( r_{\text{ mantle } } k\right) -r_{\text{ mantle } } k J_{1 / 2}\left( r_{\text{ mantle } } k\right) }, \end{aligned}$$

and $J_{\nu }(z)$ is the Bessel function of the first kind and order $\nu$, and the complex wavevector $k = (1 + i) \sqrt{\mu _0 \sigma \omega /2}$ (Parkinson 1983). Finally, the induced magnetic field at a position $\vec{r} = r \hat{r}$, measured relative to the center of the moon, is

19 $$\begin{aligned} \vec {B_s} = - A e^{-i (\omega t-\phi )} B_p [3(\vec {r} \cdot \vec {e_0}) \vec {r} -r^2\vec {e_0}] \dfrac{r_\textrm{moon}^2}{2r^5}. \end{aligned}$$

For a perfectly conducting layer, that is $\sigma \rightarrow \sigma _{\infty }$, the complex amplitude of the induced field approaches $A \rightarrow (r_\textrm{ocean}/r_\textrm{moon})^3$, and there is no phase lag, such that $\Phi \rightarrow 0$. In contrast, for finite conductivity, the amplitude of the induced field is smaller than that of the inducing field and a phase lag develops, so that A<1 and $-\pi /2< \Phi < 0$.

#### Conducting Sphere

Similarly, an analytical solution exists in the case of a sphere with homogeneous conductivity, $\sigma _\textrm{cond}$, and radius, $r_\textrm{cond}$. For Mercury, this conducting sphere is assumed to be overlain by an insulating layer such that the total planetary radius is $r_\textrm{planet}$.

Ensuring continuity of internal and external fields across $r_\textrm{cond}$, the relationship between Gauss coefficients of internal and external origin is

20 $$\begin{aligned} \dfrac{\epsilon _n^m }{\iota _n^m } = \dfrac{n}{n+1} q^{2n+1} \left( 1-\mu \left\{ \dfrac{kr_\textrm{cond}}{2n+1} \dfrac{I_{n-1/2}(kr_\textrm{cond})}{I_{n+1/2}(kr_\textrm{cond})}\right\} ^{-1}\right) , \end{aligned}$$

where $$q = \dfrac{r_\textrm{cond}}{r}$$ (Rikitake 2012).

For cases in which the argument $\vert k r_\textrm{cond} \vert$ of the modified spherical Bessel function is large, $I_{n \pm 1/2}$ can be Taylor expanded (Rikitake 2012; Grosser et al. 2004) and the following approximation can be made:

21 $$\begin{aligned} \dfrac{\epsilon _n^m }{\iota _n^m } =\dfrac{n}{n+1} \left( \dfrac{r_\textrm{cond}}{r} \right) ^{2n+1} \end{aligned}$$

Here, the ratio of inducing and induced field is independent of time and is mainly driven by the radius of the conductive core.

### Response Functions

Transfer or response functions represent a connection between an input, here the inducing field, and an output, the induced field. Generally, such response functions can be inverted to derive conductivity profiles with depth and I refer the reader to another review and references therein on inversion strategies (Grayver 2024), but focus on approaches for the derivation of response functions.

#### Global Response Functions

In cases where separation between inducing and induced fields is possible, e.g., where Gauss coefficients can be evaluated (Sect. 2.2), Eqs. 1 and 2 show that the magnetic and electric fields are linear with respect to the current $j_\textrm{ext}$, and one can thus treat the planetary body as a linear system. Assuming a radially symmetric (1D) conductivity structure, each external coefficient induces only one internal coefficient and their complex-valued ratio, the so-called *Q*-response is defined by (Schmucker 1985),

22 $$\begin{aligned} Q_n(\omega ;\sigma ) = \dfrac{\iota _n^m(\omega ;\sigma )}{\epsilon _n^m(\omega ).} \end{aligned}$$

Note that in the case of a 3D conductivity distribution, each external coefficient induces a range of internal coefficients, and *Q* needs to be described by a matrix (Olsen 1999). In the 1D case and from Eq. 22, one can formulate the complex-valued *C*-response, where the real part can intuitively be understood as the penetration depth:

23 $$\begin{aligned} C_n(r)= \dfrac{r}{n+1} \dfrac{\left( \dfrac{r}{a}\right) ^{2n+1}-\dfrac{n+1}{n}Q_n}{\left( \dfrac{r}{a}\right) ^{2n+1}+Q_n} \end{aligned}$$

Typically for the terrestrial case, the *C*-response can be computed for n=1, as the n,m = 1,0 geometry describes the dominant inducing field, which results from the ring current (e.g., Olsen 1999). For other planetary bodies, the geometry of the inducing field needs to be addressed individually, and in some cases may not be described by a single (or even a few) low degree and order spherical harmonic terms. Global response functions are a useful tool to evaluate satellite data because the effects of space and time can be separated.

#### Local Response Functions

In cases where the induced field is measured by a fixed station, the transfer function can be linked to a single location, $\vec {r} = (a,\theta ,\phi )$ for the magnetic field components $\textbf{B}(\vec {r},\omega ; \sigma )$ and the equivalent to Eq. 23 for both horizontal components is:

24 $$\begin{aligned} C_{n,\phi }(a,\theta ,\phi ;\sigma )= & \dfrac{1}{sin(\theta )}\dfrac{a}{n(n+1)} \dfrac{\partial _\phi (P^{\vert m \vert }_n (\cos \theta ) e^{im\phi })}{P^{\vert m \vert }_n (\cos \theta ) e^{im\phi }} \dfrac{B_r(a,\theta ,\phi , \omega ;\sigma )}{B_{\phi }(a,\theta ,\phi , \omega ;\sigma )} \end{aligned}$$

25 $$\begin{aligned} C_{n,\theta }(a,\theta ,\phi ;\sigma )= & \dfrac{a}{n(n+1)} \dfrac{\partial _\theta (P^{\vert m \vert }_n (\cos \theta ) e^{im\phi })}{P^{\vert m \vert }_n (\cos \theta ) e^{im\phi }} \dfrac{B_r(a,\theta ,\phi , \omega ;\sigma )}{B_{\theta }(a,\theta ,\phi , \omega ;\sigma )} \end{aligned}$$

Using these response functions, particularly in terrestrial applications, where n=1, m=0, the approach is commonly referred to as the Z/H method (Banks 1969). It is assumed that the inducing field is driven by an external source that can be represented by a single spherical harmonic function. However, if this assumption is violated, it can lead to artifacts in the derived conductivity structure (see the discussion in Grayver (2024)). Therefore, it is important to carefully evaluate the geometry of the source fields.

#### Multiple Spacecraft Transfer Function

Early Apollo literature used another procedure to derive a response function (e.g., Sonett et al. (1971), Hobbs et al. (1983)). This approach to separate inducing and induced fields requires two independent spacecraft measurements to evaluate a transfer function. Here, one measurement is recorded close to or on the ground, representing a combination of the inducing field and the induced response, the other one from a distant spacecraft in orbit recording only the inducing field. In the frequency domain, transfer function *T* is

26 $$\begin{aligned} T_i(\omega ;\sigma ) = \dfrac{B_{i,\textrm{surf}}(\omega ;\sigma )}{B_{i,\textrm{dist}}(\omega )} \end{aligned}$$

where *i* represents all three field components. Among these, *r* denotes the radial component, while *h* refers to either of the horizontal components. The transfer functions are related to apparent resistivity as

27 $$\begin{aligned} \rho _a = \omega \mu _0 \left( \dfrac{aT_r(\omega )}{T_h(\omega )}\right) ^2. \end{aligned}$$

An alternative to this frequency-domain approach was introduced by Dyal and Parkin 1971, 1973), focusing on the time-domain behavior of a conducting sphere subjected to transient external magnetic fields. Based on the theoretical principles outlined by Smythe (1950), this approach examines the global poloidal induction response generated by sudden changes in the interplanetary magnetic field associated with solar wind dynamics.

Although the multiple spacecraft approach was not derived under the assumption of a potential field, it is only suited for planetary bodies for which the distal and surface measurements show coherence, and generally, if currents are present between those sources, the signal-to-noise ratio is likely insufficient for robust determination of apparent resistivity.

## Mission Data Sets

Because mission data drive progress, I briefly review available magnetic field data and some of their characteristics, which are also summarized in Table 1 and Fig. 1. Here, I will describe existing mission data and I will come back to upcoming missions and data sets in the subsequent sections. Generally, most missions equipped with vector magnetometers, i.e., data that might be used for EM induction studies, are orbiters. Currently, exceptions only exist for Mars and the Moon.

**Fig. 4:**
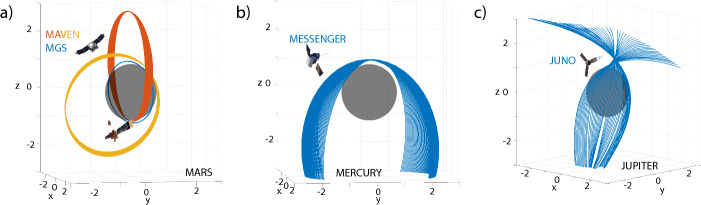
Example orbits for **a** Mars MGS from 2005-05-24 to 2005-06-04 (blue) MAVEN 2015-09-05 to 2015-09-15 (red) and 2017-09-05 to 2017-09-15 (yellow) **b** Mercury MESSENGER 2013-03-18 to 2013-04-18 (blue), **c** Jupiter JUNO 2016-08-01 to 2024-01-12 (blue). The axes are normalized by the respective planetary radii

### Mercury

Mercury has received much attention with the Mercury Surface, Space Environment, Geochemistry, and Ranging, the MESSENGER mission. MESSENGER was the first mission to orbit Mercury until it crashed into the body in 2015 (Solomon and Byrne 2019). It enabled full characterization of the external field environment (Korth et al. 2018; Slavin et al. 2018), as well as the internal field (Johnson et al. 2018) that comprises contributions from the hermean dynamo (Anderson et al. 2011), crustal fields (Johnson et al. 2015), and fields induced due to Mercury’s eccentric orbit around the Sun (Johnson et al. 2016). MESSENGER was in a highly eccentric orbit about Mercury, and so the internal field is only characterized by observations over the northern hemisphere (Fig. 4b). In particular, mapping of the crustal field is only possible for latitudes north of 30$^\circ$N, and this orbital geometry complicates magnetic sounding studies, due to limited latitudinal coverage. Note that terrestrial sounding studies avoid using data collected close to the poles - which in the case of Mercury is where the best low-altitude coverage exists.

The upcoming BepiColombo mission, a double-spacecraft mission launched by ESA and JAXA, will address these limitations. With its improved coverage, in particular southern hemisphere observations, and the combined capabilities of the Mercury Planetary Orbiter (MPO) and the Mercury Magnetospheric Orbiter (MMO), BepiColombo will contribute global measurements of Mercury’s magnetic field, surface, and interior from an orbit reaching altitudes less than $250$ km above the planet within its extended mission plan (Benkhoff et al. 2021).

### Venus

Two orbital missions with magnetometers have orbited Venus: Venus Express (Svedhem et al. 2009) and Pioneer Venus Orbiter (Russell et al. 1979). Both missions were in a polar, elliptical orbit, allowing studies to cover a wide range of altitudes, from the deep magnetosphere to the upper atmosphere. Venus Express covered a comparably wider range of altitudes with its periapsis in the northern polar region, enabling the study of global magnetospheric dynamics, while Pioneer Venus had the potential for more low altitude equatorial investigations.

### Moon

Multiple Apollo missions carried geophysical instruments, including magnetometers (Dyal et al. 1974). Apollo 12, in particular, was used for magnetic sounding of the lunar mantle. This is because with concurrent satellite measurements from Explorer 35, a two-spacecraft transfer function approach (Section 2.4.3) was possible (Sonett et al. 1972; Hood et al. 1999). Because the Explorer 35 magnetometer was degrading, such analyses were generally not feasible with later orbital data measured during Apollo 15 and 16 (Daily and Dyal 1979).

Later, satellites Lunar Prospector (Binder 1998) and Kaguya Selene (Sasaki et al. 2009), provided valuable data for multiple years in circular orbits with full coverage down to $\sim$20 km above the surface. Those were used to study the core size (e.g., Hood et al. 1999; Shimizu et al. 2013) and mantle structure (e.g., Mittelholz et al. 2021), as well as to characterize the lunar crustal fields (e.g., Ravat et al. 2020; Hood et al. 2021). Kaguya Selene operated at a time of low solar activity compared to Lunar Prospector, and some authors preferred to use individual data sets only (e.g., Ravat et al. 2020).

ARTEMIS THEMIS is a mission that includes several orbiters of which two, THB and THC, were redirected to the Moon (Angelopoulos 2014). Those missions enable the characterization of the external field environment of the Moon and have recently been analyzed in combination with the Korean Pathfinder Lunar Orbiter in a circular 100 km altitude orbit around the Moon (Jeon et al. 2024).

One recently completed and one upcoming mission include the Lunar Magnetotelluric Sounder (LMS) missions enabling the first extraterrestrial MT studies as part of one of the ongoing NASA initiatives, Commercial Lunar Payload Services (CLPS). The industrial involvement facilitated by CLPS has invigorated Moon exploration with multiple upcoming missions.

### Mars

Mars has one of the best studied magnetic field environments of planets other than Earth. The Mars Global Surveyor (MGS, 1997–2006) and Mars Atmosphere and Volatile Evolution (MAVEN, since 2014) missions have characterized the field in detail.

While MGS spent most of its time in a polar 2 am/2 pm circular orbit at $\sim$400 km, the so-called mapping orbit (Acuña et al. 1999), MAVEN has an elliptically precessing orbit (Jakosky et al. (2015); Fig. 4a). Hence, MGS provides thorough coverage at the mapping orbit and this enables stacking of data though without broad local time coverage, i.e., containing mostly zonal information. MAVEN, on the other hand, is uniquely suited for understanding the full extent of the martian magnetosphere, at variable local times and altitudes (Gao et al. 2024). It has also helped improve the description of the crustal field due to low-altitude passes during its periapsis (Mitteholz and Johnson 2022; Langlais et al. 2019). The Tianwen-1 data have additionally allowed concurrent observations to improve understanding of the dynamic magnetosphere since 2021 (Cheng et al. 2023).

NASA’s InSight lander (2018–2022) and China’s Zhurong rover (2021–2022) provide data from the surface. While Zhurong measured the horizontal magnetic field for 16 short time frames ($\sim$30 min) (Du et al. 2023), InSight provides 729 sols of continuous data and an additional Mars year of interrupted data (Mittelholz et al. 2023). However, InSight was not a science instrument, i.e., no scientific objectives were formally associated with the magnetometer, and therefore the associated cleanliness program was limited. As a result, signal contamination from the lander introduces artifacts into the measurements, particularly at diurnal periods, due to unaccounted for effects from solar array currents and temperature variations (Mittelholz et al. 2020). For Zhurong, the lack of pre-mission calibration leads to information on the horizontal field only and a calibration that relies on assumptions about the Martian ionosphere (Du et al. 2023).

### The Jupiter System

For the outer gas giants Saturn, Jupiter, and their moons some data are already available, and further missions have been launched reaching the Jovian system in 2030/2031. Typically missions to outer Solar System bodies collect data from planetary flybys during cruise, with these encounters primarily serving as gravitational assists to adjust the spacecraft’s trajectory.

**Fig. 5:**
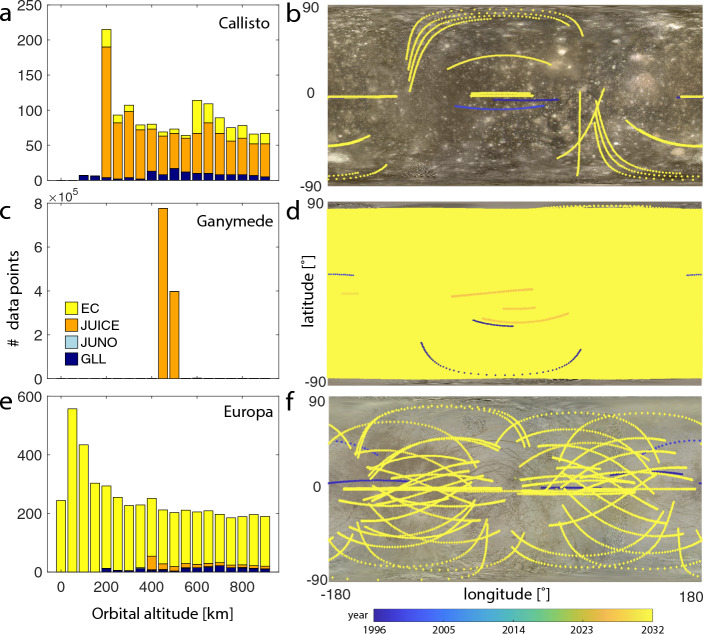
**a** Data coverage within 1000 km altitude of the surface of **a**, **b** Callisto, **c**, **d** Ganymede and **e**, **f** Europa for the Galileo (GLL), Juno, JUICE and Europa Clipper (EC) missions using spice kernel reconstructions and predictions. Galileo data cover the three main mission phases from 07/1995–09/2003, Juno from 07/2016–01/2024, while JUICE and EC show planned trajectories up to 10/2035 and up to 09/2034, respectively. **a**, **c**, **e** Histograms of number of data for each mission at altitudes less than 1000 km. Trajectories are predicted at the same sampling rate (1 sample per 10 s) to compare data coverage among missions; actual instrument data rates may differ. **b**, **d**, **f** Geographical data coverage color-coded by time

As the first mission in orbit around Jupiter, Galileo provided evidence of induction signals attributed to subsurface oceans of the Galilean moons Io, Europa, Callisto, and Ganymede (Kivelson et al. 1992). After its interplanetary cruise, Galileo entered three mission phases, the primary tour (1995–1998), the Galileo Europa Mission (1998–2000), and finally, the Galileo Millenium Mission (2000–2003), all focused on studying Jupiter and its moons with a focus on Europa in the second mission phase (Fig. 5).

The Juno spacecraft entered a polar orbit around Jupiter in 2016 and also performed several flybys of the Galilean moons. Juno has enabled detailed studies of Jupiter’s magnetic field environment, providing data that have facilitated the development of comprehensive global models of its complex magnetosphere and internal dynamo processes (Connerney et al. (2017); Fig. 4c). Juno is currently planned to operate until 2035.

In addition, ESA’s Jupiter’s Icy Moon Explorer (JUICE) and NASA’s Europa Clipper are on their way to the Jovian system (Fig. 5). While JUICE is planned to enter a circular orbit around Ganymede in addition to flybys of the other Galilean moons, Europa Clipper focuses on Europa, with flybys only 25 km above the Moon’s surface. Orbiting Europa is not feasible with the current Europa Clipper mission design due to the extreme radiation environment in its vicinity.

### The Saturn System

Cassini was a dedicated Saturn mission that operated for 13 years until it ended with the “grand finale,” where the spacecraft was steered into Saturn’s atmosphere to avoid contamination of Saturn’s moons (Spilker 2019). Cassini collected information on the magnetic field and plasma environment around Saturn, while multiple flybys of Saturn’s moons provided information on those intriguing worlds. Particularly Titan with its lake systems and Enceladus with plumes emanating from a subsurface ocean hint at active geological processes and potential chemical energy sources. Even though Cassini’s primary focus was Saturn, its Jupiter flybys provided valuable insights into the gas giant’s magnetosphere and atmospheric composition. This data complements findings from dedicated Jupiter missions such as Galileo and Juno.

### Beyond Saturn

Lastly, the little data on the magnetic fields of Uranus and Neptune have been gathered through observations made by Voyager II flybys in the 1980s (Ness et al. 1986, 1989). Both planets were confirmed to host a complex internal dynamo field. Thus, only a single flyby provides the only magnetic field data for the outermost planets Uranus and Neptune, and their moons. Voyager launched in 1977 and performed those flyby’s in 1986 and 1989. Voyager I and II have since been retasked and are now operating as the “Voyager Interstellar Mission” reaching interstellar space as the farthest traveled mission so far. With the recommendation of a Uranus orbiter Flagship Mission in the last decadal survey, NASA has declared the outer solar system as a future prime target for exploration (National Academies of Sciences 2023). Similarly, ESA has announced Enceladus and/or Titan as critical targets in their long-term planning report “Voyage 2050” (Buonanno et al. 2021).

**Table 1:** Missions with magnetometers in orbit or on the surface of planetary bodies. Mission duration is listed as orbital insertion or landing of the spacecraft (not mission launch) if not noted otherwise. Planned missions are included where relevant

Body	Mission	Mission characteristics
Mercury	MESSENGER (Anderson et al. 2011)	Highly elliptic orbit with orbital period of $\sim$12 h; in orbit: 2011–2015
	BepiColombo (Heyner et al. 2021)	Double-spacecraft mission (planned orbit insertion: Dec 2025)
Venus	Pioneer Venus (Russell et al. 1979)	Elliptical orbit, inclination 105$^{\circ }$ ($180 \times 67$,000 km) of 24 hr period (1978–1992)
	Venus Express (Svedhem et al. 2009)	Elliptical polar orbit (250–$350 \times 66$,000 km), orbit of 24 hr period (2006–2014)
Moon	Apollo 12	Lander with Lunar Surface Magnetometer (LSM) at 3$^{\circ }$S, 23$^{\circ }$W (1969)
	Apollo 15	Lander with LSM at 26$^{\circ }$N, 4$^{\circ }$E (1971)
	Apollo 16 (Dyal et al. 1974)	Lander with LSM at 9$^{\circ }$S, 16$^{\circ }$E (1972)
	Explorer 35 (Sonett 1982)	Satellite in lunar orbit, 147$^{\circ }$inclination, (1966–1972); degradation of magnetometer over time
	Lunar Prospector (Hood et al. 1999)	Satellite in polar orbit with varying altitudes (1998–1999)
	Kaguya Selene (Takahashi et al. 2009)	Satellite in polar orbit with varying altitudes (2007–2009)
	ARTEMIS THEMIS (Angelopoulos 2014)	Multiple spacecraft of which two, THB and THC, were redirected to the Moon orbiting the Moon’s L2 and L1 Lagrange point, 500x16’000 km
	Korea Pathfinder Lunar Orbiter (Jeon et al. 2024)	Satellite in polar orbit at 100 km altitude (since 2022)
	Lunar Magnetotelluric Sounder - 1 (Grimm et al. 2021)	Mare Crisium at 18$^{\circ }$N and 59$^{\circ }$E (2025)
	Follow up: Lunar Magnetotelluric Sounder - 2	Schrödinger Basin at 75$^{\circ }$S and 133$^{\circ }$E (planned for 2027)
Mars	MGS (Acuña et al. 1999)	Satellite in 2am/2pm mostly circular orbit at 400 km altitude (1998–2006)
	MAVEN (Connerney et al. 2015)	Satellite variable altitudes and local times, lowest altitude coverage $\sim$150 km (since 2014)
	InSight (Johnson et al. 2020)	Lander at 4$^{_\circ }$N, 137$^{_\circ }$E (2018–2022)
	Zhurong (Du et al. 2023)	Rover at 25$^{\circ }$N, 110$^{\circ }$E with <1 km traverse (2021)
	Tianwen-1 (Liu et al. 2020)	Satellite mostly in $265 \times 12$,000 km altitude polar orbit (since 2021)
Jupiter and moons, notably Io, Europa, Callisto and Ganymede	Galileo (O'Neil and Mitchell 1983)	Arrival at Jupiter in 1995 several flybys of Galilean moons. Mission ended through a planned crash into Jupiter (1995–2003)
	Juno (Bolton et al. 2017)	Eccentric polar orbit $4200 \times 75$,600 km. Orbital insertion: 2016. Flyby’s of Ganymede (2021), Europa (2022) and Io (2023 and 2024). Planned end of mission: 2035
	JUICE (Grasset et al. 2013)	Launched in 2023; Ganymede flyby and Jupiter orbit insertion (2031); Europa flyby (2032), elliptical orbit around Ganymede (2034); circular orbit at 500 km altitude (2035). Planned end of mission: 2035
	Europa Clipper (Roberts et al. 2023)	Launch in 2024; orbit insertion and multiple flybys of Galilean moons (2030); science at Europa with flybys at 25 km distance from surface (2031). Planned end of mission: 2034
Saturn and moons, notably Titan and Enceladus	Cassini (Dougherty et al. 2005)	Strongly eccentric orbit with multiple flyby’s of different moons (in orbit: 2004–2017) other: Jupiter flyby’s in 2020. The mission ended with the “Grand Finale”, a planned crash into Saturn

## External Magnetic Field Environments

Planetary magnetic field environments govern the degree of interaction between the solar wind with planetary bodies and their atmospheres and thus the degree to which currents are induced in their interior. In addition, but not the focus of this review, it strongly influences the degree to which and how atmospheres can escape and turn a planet into a potentially habitable world or not (Ramstad and Barabash 2021).

**Fig. 6:**
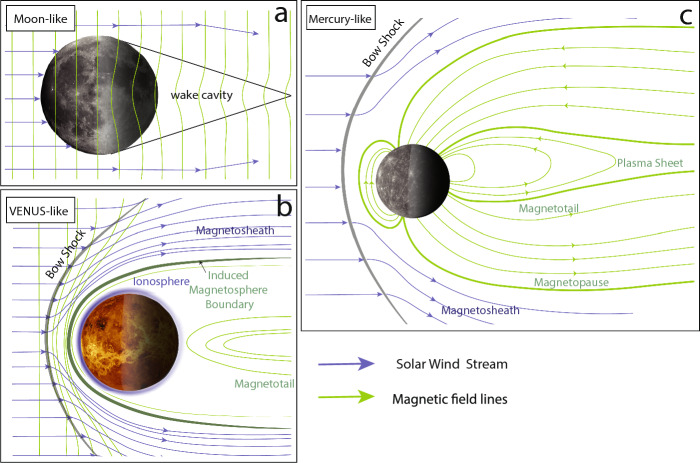
Solar wind interactions of Moon-, Venus- and Mercury-like bodies. For all panels the Sun is on the left. The IMF magnetic field lines are shown in the Moon- and Venus-like cases. For the Mercury-like case only field lines inside the magnetosphere are shown. The wake cavity in the Moon-like case describes the region in the shadow of the planet/moon which is characterized by reduced fields.

Planetary magnetospheres are defined as the region around a planetary body in which the body itself influences the behavior of charged particles in its vicinity (e.g., Luhmann et al. 2004; Baumjohann et al. 2010; Kivelson and Bagenal 2014). In some cases this region extends to many planetary radii at which point the magnetosphere is confined by the solar wind, a low density magnetized plasma that flows radially outward from the Sun. The magnetic field that is produced by the Sun is frozen into the highly conducting plasma and spirals around the Sun due to solar rotation. This is the interplanetary magnetic field (IMF) that interacts with planets in the Solar System.

Planetary bodies without intrinsic magnetic fields or atmosphere are directly exposed to the solar wind and represent one end-member of magnetospheric interaction. This is the case for the Moon for which the solar wind interacts directly with the surface, is absorbed, and creates a wake cavity devoid of plasma downstream (Fig. 6 Moon-like). Note that while evidence for an ancient lunar dynamo field exists through remanent magnetic fields detected in crustal rocks (Weiss and Tikoo 2014; Tikoo et al. 2017), those fields are weak and do not significantly contribute to deflecting external magnetic fields. The other end members are planets with an intrinsic global magnetic field that interacts with the solar wind; this is the case for Earth or Mercury (Fig. 6 Mercury-like). On Earth, the internal dipole field is the dominant contribution to the magnetic field environment. Mercury’s internal field is much weaker, leading to a much more compressed dayside field. During high solar activity, the solar wind can intermittently interact directly with the surface (Johnson et al. 2016). The in-between case is a magnetosphere of “unmagnetized” bodies, those lacking an internal dynamo field, but possessing ionospheres such as Venus or Mars (Fig. 6 Venus-like). While no crustal fields have been detected on Venus to date (Phillips and Russell 1987), Mars exhibits locally strong crustal fields. These local magnetic fields can stand off the solar wind (similar to a global dipole field), forming so-called “mini-magnetospheres” that can extend up to more than 1000 km (Crider et al. 2001; Fan et al. 2023).

When the solar wind approaches Venus or Mercury-like planets, it gets decelerated from supersonic to subsonic velocities at the bow shock due to the planetary obstacle (Fig. 6 Venus-like). Piled-up field lines and compressed solar wind characterize the magnetosheath region below. The magnetopause represents the boundary at which the solar wind pressure is balanced by the intrinsic magnetic field pressure for planets with a dynamo field. The ionopause or induced magnetosphere boundary is the analogous boundary for planets without intrinsic dynamo fields but with an ionosphere (i.e., Venus-like). Stretched-out field lines behind the planet form the magnetotail (Luhmann et al. 2004).

Generally, the size of the magnetosphere of a planet depends on its radius and magnetic field in addition to the ambient solar wind density, which falls off as the inverse square of the distance from the Sun. This is important in the context of EM sounding because it determines to which degree the Sun itself is a driver of inducing fields or if the inducing field can be purely described by a planetary magnetic field. This is, for example, the case for the Jovian moons for which Jupiter itself is the dominant source of inducing fields. Also, despite Uranus’ and Neptune’s small fields compared to the gas giants, their magnetosphere size is large (Table 2) due to the distance from the Sun and corresponding decreased solar wind density.

**Table 2:** Magnetospheres of planets with active dynamos (Kivelson and Bagenal 2014)

	Mercury	Earth	Jupiter	Saturn	Uranus	Neptune
Magnetic moment ($M_\textrm{Earth}$)	4 $\times 10^{-4}$	1	20,000	600	50	25
Size of magnetosphere ($R_\textrm{planet}$)	1.5 $R_M$	8–12 $R_E$	63–93 $R_J$	22–27 $R_S$	18 $R_U$	23–26 $R_N$

## EM Sounding Studies

Equipped with an understanding of inducing field environments, available data and methods, I will next discuss each planetary body individually. The extent of available literature, and hence the detail in discussion, depends largely on the extent of available data.

### Mercury

#### The Magnetic Field Environment

Following the Mariner flybys in the 1970s (Ness et al. 1974), MESSENGER was the first spacecraft in orbit that allowed comprehensive investigation of Mercury’s dynamic magnetic field environment (Anderson et al. 2008; Johnson et al. 2012; Wardinski et al. 2019). Although the presence of a predominantly dipolar intrinsic magnetic field suggests a field environment similar to that of Earth, there are significant differences (Slavin and Holzer 1979; Korth et al. 2018). Because Mercury is close to the Sun, the solar wind ram pressure is $\sim$10–30 nPa as opposed to $\sim$2 nPa at Earth and the IMF magnitude is $\sim$30 nT, as opposed to $\sim$5 nT (Korth et al. 2011). In addition, the planetary magnetic moment of Mercury is weak, $\sim$190 nT $R_\textrm{Merc}^3$, corresponding to a dipole moment that is smaller by a factor of about 1000 compared to Earth’s (Johnson et al. 2012). This leads to a magnetosphere that is a factor of 7–8 times smaller than that around Earth and the stand-off distance of Mercury’s Sun-facing magnetopause is approximately 1.4 to 1.5 $R_\textrm{Merc}$ from Mercury’s offset dipole (Winslow et al. 2013; Zhong et al. 2015; Philpott et al. 2020) varying with heliocentric distance. Furthermore, timescales for convection and waves transiting the magnetosphere are almost two orders of magnitude faster (Slavin et al. 2018).

The global-scale dynamo field is generated in Mercury’s liquid outer core. Several models can reproduce observations of a weak active dynamo field, for example, due to stably stratified layers or precipitation of iron (“iron snow”) (Christensen and Wicht 2008; Manglik et al. 2010; Vilim et al. 2010), although explaining the north/south hemispheric asymmetry is more difficult (see review in Johnson et al. (2018)). MESSENGER data also revealed weak crustal fields (Johnson et al. 2015) only visible near the crustal source region. This observation was possible because the MESSENGER orbit was lowered for an eventual impact that ended the mission.

Because the internal field contribution is generally weak, externally generated fields substantially influence the observed magnetic field signal at orbital altitudes (Johnson et al. 2018; Korth et al. 2018). A classic spherical harmonic analysis has been applied to separate internal and external field components (Wardinski et al. 2019); this approach relies on a potential field assumption and is therefore only applicable in source-free regions. However, at Mercury a potential field assumption is problematic because currents are observed throughout the magnetosphere including close to the surface. Further, field-aligned, so-called Birkeland currents, have been found to increase in amplitude with decreasing altitude (Anderson et al. 2014). Toepfer et al. (2021) investigated this further by extending the Gauss representation to a toroidal-poloidal field decomposition, a ‘Gauss-Mie’ representation, which accommodates the presence of current systems and non-potential fields in the magnetosphere. This extension allows for a more comprehensive characterization of the magnetic field by incorporating both the divergence-free (poloidal) and curl-free (toroidal) components. In doing so, Toepfer et al. (2021) provided a framework for better distinguishing contributions of internal, induced, and external fields, even in regions where current systems are active, such as near the surface or within the magnetosphere.

Alternatively, a model to describe the external field environment is an empirically parameterized model (Korth et al. 2015, 2017). Here, the internal field is represented by an internal dynamo field (190 nT $R_\textrm{Merc}^3$) offset by 479 km along the spin axis and the geometry and strength of the tail current sheet and magnetopause are defined. The geometry of the tail and magnetopause current systems is parametrized and constrained from observations of the magnetopause and tail current sheet locations. The magnetospheric fields are then calculated under the assumption that the net normal magnetic field component across the magnetopause is zero (Johnson et al. 2012; Korth et al. 2015, 2017). The most recent of such models by Korth et al. (2017) is based on the static version of the model (Korth et al. 2015) but takes into account the dynamic nature of the magnetosphere on time scales of MESSENGER’s orbital period and longer, by parameterizing the magnetospheric current systems as a function of heliospheric distance and magnetic activity.

#### Conductivity Structure

Electromagnetic sounding of Mercury represents a unique situation due to its large conductive core overlain by a comparably thin and insulating mantle. Because the mantle is so thin and highly resistive, only high frequencies would probe the mantle, and this has not been attempted so far. Typical geomagnetic depth sounding approaches (Olsen 1999) assuming Earth-like geometries might be a feasible option in the future, but using MESSENGER data it is extremely challenging because of the highly elliptical orbit with its periapsis above the north pole and hardly any data coverage at mid-latitudes (Fig. 4b).

Therefore, EM studies for Mercury have focused on addressing the size of its large core. Such studies rely on the fact that time-varying magnetic fields penetrate the core inducing a magnetic field. The characteristic time scale for the external field to diffuse into the core overlain by a mantle can be approximated as $T_D = \mu \mu _0 \sigma L^2$, where $\mu =1$. Assuming $L=400$ km for the resistive outer mantle layer with $\sigma = 10^{-2}$ S/m, this results in $T_D \sim 1$ h. Therefore, variations with durations longer than approximately one hour penetrate into the core, and on those time scales, one can ignore induction in the resistive mantle and crust (Suess and Goldstein 1979).

**Fig. 7:**
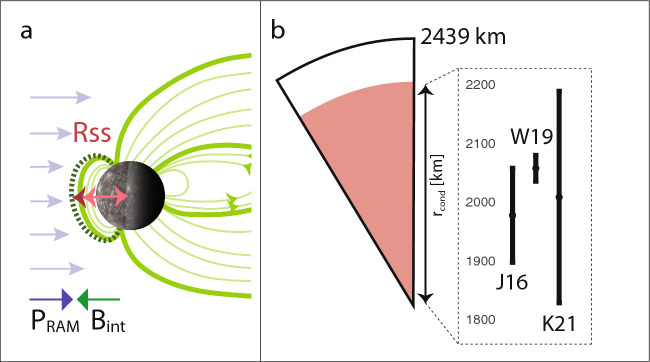
**a** Sketch of the influence of eccentricity on solar wind pressure $P_\textrm{ram}$ and the oppositely directed internal field $B_\textrm{int}$ **b** The interior structure of Mercury in a two-layer model and $r_\textrm{cond}$ estimates from three separate EM induction studies J16, W19 and K21 (Johnson et al. 2016; Wardinski et al. 2019; Katsura et al. 2021)

This justifies the use of the conductive sphere model with a highly conductive core covered by an insulating mantle (Sect. 2.3.2). Because it is unclear whether the transition from resistive to conductive corresponds to the core or an overlying metal-rich layer, I, like many authors, refer to $r_\textrm{cond}$ as the radius of the highly conductive layer, possibly the core. Note that the relationship between the inducing and induced fields for any given spherical harmonic degree *n* is then solely a function of the radius of the conductive layer. Several studies have used this approach to estimate $r_\textrm{cond}$ using MESSENGER, with different strategies for representing the inducing and induced fields described in the following. Resulting $r_\textrm{cond}$ are compared in Fig. 7b.

Johnson et al. (2016) used the fact that the standoff distance of the magnetopause ($R_\textrm{SS}$) varies with solar wind pressure, which itself depends on heliospheric distance, $r_h$. Compression of the magnetopause due to increased solar wind pressure induces currents in the planetary interior. These currents generate magnetic fields that oppose the compression, resulting in a magnetopause location ($R_\textrm{SS}$) that is smaller than would be expected if there was no induction. In the absence of induction, $R_\textrm{SS} \sim r_h^{1/3}$; a weaker dependence (i.e., exponent $n < 1/3$) indicates the presence of induction, which reduces the compressibility of the magnetosphere (Fig. 7a). Johnson et al. (2016) found that $R_\textrm{SS}$ does indeed scale with $r_h$ with an exponent $n < 1/3$, confirming induction effects. Additionally, they observed an annual modulation in the internal magnetic field measured within the magnetosphere, consistent with variable induction driven by Mercury’s elliptical orbit. Together, these two independent observations of induction from 15 Mercury years of data yielded an annual variation in the internal dipole term $g_1^0$ of 7.5–9.5 nT. This study provided the first data-based evidence for a measurable effect of induction at Mercury. In addition, Johnson et al. (2016) used the parameterized model of Korth et al. (2015) to evaluate the spherical harmonic coefficients associated with different magnetopause standoff distances ($R_\textrm{SS}$), thereby deriving the inducing field. Using a two-layer model (Eq. 21), they then constrained $r_\textrm{cond}$ by matching the predicted and observed induced fields. They obtained $r_\textrm{cond}$ = 1900–2060 km, the first non-geodetic estimate for Mercury’s core size.

Wardinski et al. (2019) used MESSENGER magnetic field data to conduct a spherical harmonic analysis, separating the internal and external magnetic fields (under the assumption of a current-free magnetosphere) up to degree 3 for a static and a time-varying field. They showed that the temporal variations between the internal and external coefficients are correlated and that, within the 5-day temporal resolution of the data, no time lag could be detected; this placed an upper limit of $\sigma _\textrm{Mantle} \sim 1$ S/m. This bound is consistent with synthetic conductivity profiles derived by Verhoeven et al. (2009) for which $\sigma _\mathrm{Mantle/Crust}$ ranges from $10^{-4}$ to 1 S/m, and pre-MESSENGER assumptions used for two-layer models (e.g., Grosser et al. 2004; Glassmeier 2000). Based on Eq. 21, Wardinski et al. (2019) further evaluated $r_\textrm{cond}$ by comparing the internal and external dipole moments, i.e., $n = 1$, resulting in $r_\textrm{cond}=2060 \pm 22$ km.

**Fig. 8:**
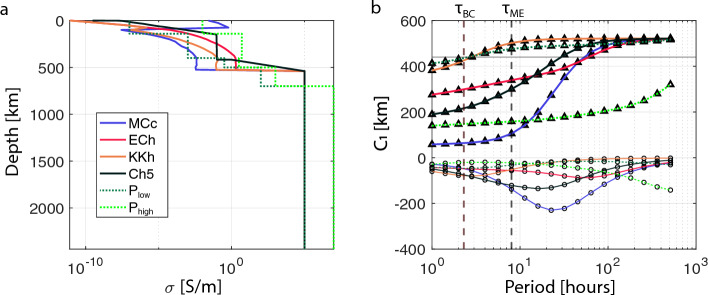
**a** Conductivity models for Mercury for a range of mineralogies indicated by MCc, ECh and KKh by Verhoeven et al. (2009). The Ch5 profile is from Anderson et al. (2018). $P_\textrm{low}$ and $P_\textrm{high}$ are lower and upper bounds from pressure experiments by Zhang and Pommier (2017). **b** Resulting complex $C_1$ response from corresponding profiles in (**a**) using the recursive formula from Kuvshinov and Semenov (2012). Triangles represent the real and circles the imaginary part. Vertical dashed lines mark the orbital period of BepiColombo (BC) and MESSENGER (ME)

Also, relying on variations in $R_\textrm{SS}$ and thus a Mercury year and its first harmonic, Katsura et al. (2021) used a parameterized external field model as an inducing time series (Korth et al. 2015) and fit zonal internal Gauss coefficients up to degree four to each individual year of MESSENGER data for the internal magnetic field description. They used fits to the time series to derive the ratio of induced vs. inducing field for each year and up to degree 4 (Eq. 21) leading to $r_\textrm{cond}=2011 \pm 180$ km. Note that this reported result excludes the ratios derived using the period of 1/2 Mercury year, which generally led to $r_\textrm{cond}$ smaller than what the authors deemed reasonable. They also attempted to fit non-zonal terms and used their own external field model by fitting external Gauss coefficients; both attempts are described as “unstable for certain data” and were not further pursued.

A conductivity range of 0.01–1 S/m for the lowermost mantle was suggested by Verhoeven et al. (2009) based on different compositions, thermal profiles and electrical conductivity estimates for each mineralogical phase. However, MESSENGER postdated this investigation and indicated a thinner crustal and silicate layer than what was assumed in this study. A later laboratory-based study provides further constraints on conductivity structure taking into account MESSENGER findings (Zhang and Pommier 2017). Their radial conductivity profile was derived from multi-anvil pressure experiments investigating metal olivine systems at 5 and 6 GPa and up to 1675$^{\circ }$C. Conductivity profiles from both studies are plotted in Fig. 8a.

#### Outlook

BepiColombo is an ESA mission currently on its way to Mercury (Benkhoff et al. 2021). BepiColombo consists of two spacecraft, equipped to measure the magnetic field at two points simultaneously. The lower altitude Mercury planetary orbiter (MPO) will initially be inserted in a $480 \times 1500$ km orbit with an orbital period, $\tau _\textrm{BC}$ of $\sim$2.3 h allowing for multiple passes per day (Heyner et al. 2021). Note that the periapsis will be lowered over time. Taking into account a range of conductivity profiles from mentioned laboratory-based studies, I estimate resulting $C_1$ responses (Fig. 8b; Kuvshinov and Semenov (2012)) to investigate whether the period range with the lowest bound, the orbital period of MPO, will give insight into the interior structure. This assumes a dipole structure ($n=1$, $m=0$) dominating the external inducing field, as is commonly assumed for Earth-based analyses. This exercise indeed shows that multiple hot and cold models and different core sizes are detectable in the covered frequency ranges. In addition, with two spacecraft in orbit, dual spacecraft studies might open up further possibilities.

### Venus

Venus magnetic field environment is characterized by an induced magnetosphere (Fig. 6); it has an ionosphere but no dynamo field. Venus is the only planet in the Solar System for which no signs of a past or current dynamo field have been detected (Phillips and Russell 1987). Because diurnal or annual periods on Venus are extremely long, 243 and 225 Earth days, respectively, inducing fields associated with such periodicities are not practical. Thus, Grimm et al. (2012) suggest that a feasible frequency range for EM conductivity studies is greater than 1 Hz. In this range, electromagnetic waves such as Schumann resonances of 10–40 Hz excited globally by lightning or more regional high frequency pulses may probe depths of $\sim$10–100 km. Besides this one study, no further work has addressed Venus’ electrical conductivity structure and EM sounding.

### The Moon

#### Interior Structure

Geophysical data on the Moon are more abundant than for any other extraterrestrial body and have allowed constraining its interior structure. In addition to magnetic field observations, datasets include seismic, magnetic, gravity, heat flow, and topographic measurements obtained from both surface instruments, primarily from the Apollo missions, and a variety of orbital platforms. Additional insights come from lunar surface samples, which complement geophysical observations. The crust, predominantly composed of anorthitic plagioclase, is highly resistive ($\sim 10^{-9}$ S/m; Dyal et al. (1974)) with a global average thickness of 34–43 km (Wieczorek et al. 2013). There are, however, significant differences locally. Geochemical studies reveal distinct compositional variations across the surface, and, in particular, the Procellarum KREEP Terrane (PKT) exhibits enrichment in Potassium, Rare Earth Elements, and Phosphorus. While these differences should be reflected in electrical conductivity, the subsurface extent of PKT is not well constrained (Grimm 2013). Locally, crustal fields have been observed from orbit (e.g., Ravat et al. 2020), and, in combination with magnetized Apollo samples (Tikoo et al. 2017; Weiss and Tikoo 2014), indicate episodes of an ancient lunar dynamo. Yet, the extent to which near-surface compositional and magnetic heterogeneities persist at depth is still unknown (Wieczorek et al. 2006; Khan et al. 2013).

**Fig. 9:**
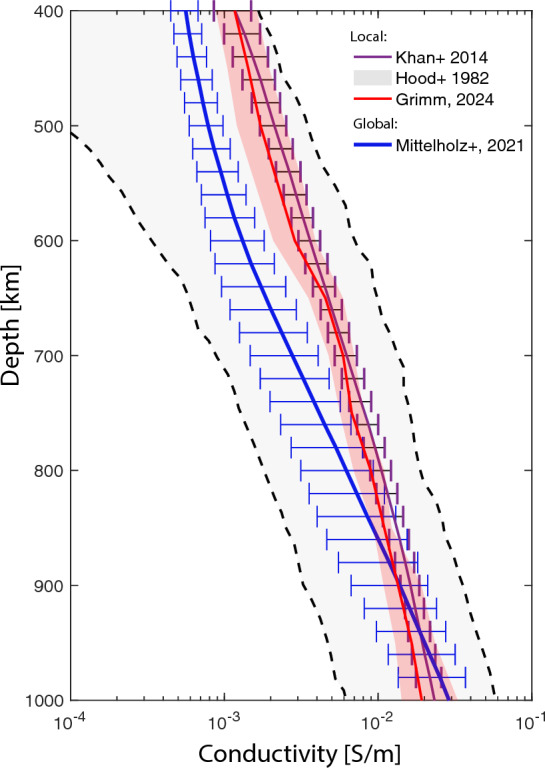
Conductivity profiles for the lunar mantle. Note that the profile by Grimm (2024) and Khan et al. (2014) use an Apollo 12 derived transfer function by Hood et al. (1982). Mittelholz et al. (2021) use a satellite derived transfer function and the same compositional model as Khan et al. (2014).

The lunar mantle is composed predominantly of olivine and pyroxene, and several mantle discontinuities have been proposed (Khan et al. 2013). Electrical conductivity of the mid and lower mantle mantle ranges between $10^{-2}$ S/m and $10^{-4}$ S/m (Fig. 9). The composition and structure of the mantle are thought to reflect the crystallization history of the Moon’s early magma ocean, which led to the formation of iron-bearing cumulates (IBCs) during its later stages. Gravitational instability of the early mantle likely drove mantle overturn (Hess and Parmentier 1995), redistributing dense IBCs. This overturn could have resulted in a stably layered structure (Elkins-Tanton et al. 2011), a compositionally heterogeneous mantle with varying amounts of IBC (Zhao et al. 2019), or a fully mixed mantle due to thermally driven convection (Zhang et al. 2013).

Finally, a dominantly iron core was suggested by several lines of evidence, including EM sounding (e.g., Hood et al. 1999; Weber et al. 2011; Garcia et al. 2011). However, the existence of a partially molten layer on top of the core is still debated. Apollo seismic data indicated a partially molten layer on top of the lunar core (Weber et al. 2011), and this was corroborated using joint inversions of mass, moment of inertia, and electrical conductivity (Khan et al. 2014). This layer could represent a titanium rich layer that formed during the lunar magma ocean and sank as part of a mantle overturn event (Elkins-Tanton et al. 2011). However, others have shown that this liquid layer is not uniquely required by the data and may even be inconsistent with some geophysical constraints (Nimmo et al. 2012; Jaumann et al. 2012). As such, uncertainties regarding the structure and composition of the core mantle boundary persist.

#### The Magnetic Field Environment

**Fig. 10:**
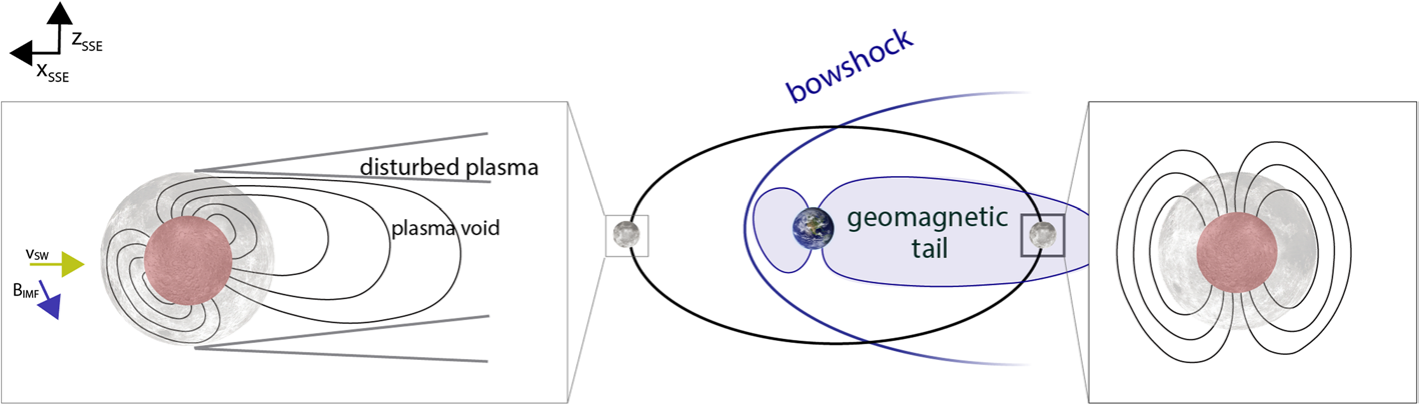
Schematic illustration after Fuqua Haviland and Mittelholz (2025) of the external magnetic field environment of the Moon orbiting the Earth. (left box) The induced field is confined within the lunar surface when the Moon is in the solar wind. (right box) A dipole-like induced response in the geotail.

As described above, the Moon's magnetic field environment represents an end-member among planetary magnetospheres, as it does not possess an ionosphere or active dynamo field (Fig. 6). The Moon thus progresses through a dynamic plasma environment within one lunation ($\sim$ 29 days) (Fig. 10). During $\sim$70% of its orbit, the Moon is directly exposed to the dynamic solar wind (Fig. 10 left) and its embedded interplanetary magnetic field (IMF), which serves as the primary driver of induction. On the dayside, solar wind and magnetosheath plasma can compress and confine weak induced magnetic fields near the surface, as the dynamic pressure of the solar wind typically exceeds the pressure of any induced magnetic response (Sonett 1982); see Fig. 10).

Absorption of plasma particles leads to a trailing plasma void of varying extent depending on solar wind activity (Halekas et al. 2005, 2010). This confinement may involve current closure through the photoelectron sheath, though such systems remain unobserved. Because the solar wind is supermagnetosonic (Colburn et al. 1967), induced signals cannot propagate upstream, further limiting detectability (Fatemi et al., 2015; Fuqua Haviland et al., 2019; Dyal and Parkin, 1971.

On the nightside, the low-density plasma wake allows induced fields to extend farther into space. Under magnetically dominated conditions, these may approximate vacuum-like dipole responses, though they can be modified by wake-associated diamagnetic and field-aligned currents (Fatemi et al. 2015; Fuqua Haviland et al. 2019).

During this time, the Moon also crosses the terrestrial bow shock, and for $\sim$4 days per lunation it travels through the terrestrial magnetosphere, including the magnetosheath and geotail (Fig. 10 right). The geotail, that is, the inducing field at that time, points away from or toward Earth, depending on whether the Moon is in the upstream or downstream geomagnetic tail. This field is dominated by large-scale structure and can be described to first order by Gauss coefficients of degree and order 1 (Mittelholz et al. 2021).

#### Conductivity Studies

Lunar magnetic sounding studies have primarily aimed to (i) constrain the lunar core size and (ii) characterize mantle conductivity and thus compositional and thermal structure. Many of those studies are based on Apollo data and were conducted in the 1970s and 1980s, as summarized in reviews on lunar EM sounding (Sonett 1982). In addition, a recent comprehensive review focused on lunar EM sounding provides more details on this topic (Fuqua Haviland and Mittelholz 2025). Here, I provide a summary on how transfer functions were derived and then describe implications of the derived findings.

***Core size***

To estimate the size of the lunar core, the lander-satellite transfer functions (Sect. 2.4.3) were initially used to obtain the upper limit of the core size (Hobbs et al. 1983; Dyal et al. 1976). Later, techniques relying exclusively on satellite data exploited the Moon’s entry into the steady terrestrial magnetotail field from the more variable solar wind and magnetosheath (Hood et al. 1999; Russell et al. 1981; Shimizu et al. 2013). This transition induces currents in the lunar interior; within the mantle, current-induced fields decay within hours, while within the conductive core they decay much slower (> 10 days). The resulting core-induced field opposes the external magnetic field change, and its signal depends on the core radius.

An induced magnetic moment of $-4.23\pm 0.64 \times 10^{23} \mathrm {Am^2T}^{-1}$ was derived using Apollo 15 and 16 subsatellites (Russell et al. 1981), resulting in a core size of approximately 400 km. The stacking of 21 Lunar Prospector orbits while the Moon was in the geotail resulted in an estimate of $-2.4\pm 1.6 \times 10^{23}\mathrm {Am^2T}^{-1}$ and a core radius of 340 ±90 km for a metallic core (Hood et al. 1999). Shimizu et al. (2013) performed a spherical harmonic expansion and separated the internal and external fields within the tail lobe using Kaguya Selene data. Assuming a geometry parallel to the tail lobe ($n=m=1$) and an insulating lunar mantle, the radial magnetic field at the core must be zero (Eq. 11). The core radius is then a function of the ratio of the internal to the external dipole field, $2 \dfrac{\epsilon _1^1}{\iota _1^1} = \left( \dfrac{r_\textrm{core}}{r_\textrm{Moon}}\right) ^3.$ Here, the mean core size was found to be 290 km with an upper bound of 400 km. Generally, such studies agree with the seismic and geodetic constraints of a lunar core size and <400 km (Nakamura et al. 1974; Weber et al. 2011; Garcia et al. 2011).

***Mantle conductivity***

During the Apollo era, multi-point transfer function analyses (Sect. 2.4.3) were enabled by the availability of magnetometers on the surface and in orbit, allowing studies of induced fields using both time-domain approaches based on transient events in the nightside wake and magnetotail lobes (Dyal and Parkin 1971; Dyal et al. 1976), and frequency-domain methods on the dayside assuming confined, quasi-stationary fields (Blank and Sill 1969; Sonett et al. 1971; Hobbs et al. 1983).

Among the first analyses, swaths of Explorer 35 and Apollo 12 surface data from the sunlit side (Sonett et al. 1971) and the dark side (Dyal and Parkin 1971) were used to derive transfer functions. Interpretation of those indicated deep layers with substantially higher conductivity ($\sigma$ $\sim$ $10^{-3}$ S/m at 800 km) than more surficial material. With increased data availability, several swaths and more than 120 hours of data were used to calculate transfer functions across 0.5–40 mHz (Sonett et al. 1972). The average and standard deviation of these were reported as a resulting composite transfer function with uncertainty, allowing sounding of mid-mantle depths. Later, Hood et al. (1982) evaluated transfer functions spanning 0.01–1 mHz, sounding the deeper mantle. These transfer functions have been used broadly and interpreted in greater detail (Khan et al. 2006, 2014; Grimm 2013, 2024). The mentioned studies all used data collected in the solar wind and sheath region and provide local conductivity estimates at the Apollo 12 landing site. The degradation of the Explorer 35 magnetometer (Daily and Dyal 1979) limited similar analysis for later Apollo surface magnetometers. Note that transfer functions from frequency-domain studies generally yielded lower conductivities than those derived from time-domain studies. This difference has generally been attributed to unmodeled wake confinement effects (Sonett 1982; Fatemi et al. 2015; Fuqua Haviland et al. 2019).

Grimm (2024) recently reanalyzed Apollo transfer functions by Hood et al. (1982) and Sonett et al. (1972) to estimate electrical conductivity for a broader depth range due to the increased frequency content of the combined transfer functions. For the low frequencies (Hood et al. 1982), he derived a 1D profile similar to a previous reanalysis by Khan et al. (2014) and within error bars of estimates from Hood et al. (1982) (Fig. 9). Using previously published temperature profiles, compositional fits for those profiles were evaluated. However, reanalysis of the higher frequency transfer functions from Sonett et al. (1972) led to high conductivities that were considered unrealistic. The author ascribed this to the effect of high frequencies because the corresponding solar wind turbulence wavelengths are on the scale of the lunar radius with increasing frequency, which might require taking into account more complex inducing environments and a multipole treatment.

In contrast to methods requiring surface and orbital data, Mittelholz et al. (2021) performed a classic geomagnetic depth sounding analysis (Sect. 2.4.1) to obtain a global *C*-response from satellite magnetic field data. Demonstrating the quasi-uniform geometry of the inducing field characterized by Gauss coefficients of degree and order 1, they derived a global *C*-response from Kaguya Selene and Lunar Prospector data in the geomagnetic tail. Although the frequency content covered does not allow shallow depth sounding and is most sensitive to $\sim$ 600–900 km, the results indicate global conductivities comparable to those obtained from local Apollo 12-based transfer functions (Fig. 9).

***Implications***

While early studies focused on broadly addressing interior structure, later studies were able to address more specific questions relying on the inversion of transfer functions, often with additional constraints on temperature or composition. Khan et al. (2006) reinterpreted lunar dayside transfer functions by Hood et al. (1982), focusing on directly estimating the chemical composition and thermal conditions using a CaO–FeO–MgO–Al2O3–SiO2 model system. By integrating laboratory conductivity data with Gibbs free energy calculations and combining these with constraints from lunar mass and moment of inertia, they performed a joint inversion to constrain interior composition and temperature. This framework was extended in Khan et al. (2014) to explore the presence of a lowermost mantle melt layer overlying the fluid core. Indeed, a partially molten zone enriched in *FeO* and $TiO_2$ was found to be necessary to fit the data, supporting magma ocean crystallization followed by cumulate overturn as a key stage in lunar evolution.

Regionally, Grimm (2013) modeled the thermal evolution of the Procellarum KREEP Terrane (PKT), linking high conductivity to localized enrichment in heat-producing elements. Their models suggested that melt or high-temperature zones in the upper mantle could have been transient and that present-day conductivity reflects past conditions rather than ongoing convection.

Furthermore, and as mentioned before, Grimm (2024) computed new profiles of electrical conductivity with depth by analyzing Apollo-Explorer transfer functions. Low-frequency data (< 1 mHz) revealed mantle temperature profiles consistent with conductive cooling and anticipated levels of iron in the mantle. The observed consistency in iron content (Mg$\#$ 81 ± 10) may reflect efficient mixing driven by ancient convection processes that are no longer active. Alternatively, incomplete overturn of unstable magma ocean cumulates might have resulted in a patchy distribution of minerals at scales too fine for electromagnetic sounding to resolve. Another interpretation suggests that the probed region represents the initial equilibrium crystallization phase within a mantle that lacked significant buoyant overturn.

While electrical methods provide important constraints on core properties, the EM signal at depth could also originate from a basal melt layer, as proposed by Weber et al. (2011). Grimm and Delory (2012) showed that although metallic and silicate cores produce distinguishable EM signatures in principle, they are nearly indistinguishable at Apollo-era frequencies. They found that a metallic core produces a characteristic flattening of apparent resistivity at low frequencies ($<10^{-5}$ Hz), whereas molten silicate cores yield continuously varying resistivity curves. Resolving this ambiguity would require lower frequency data and improved measurement accuracy that is below current mission capabilities. Therefore, while EM data support the presence of a high-conductivity feature near the core-mantle boundary, it remains unresolved whether it reflects a molten Fe-rich core, a silicate melt layer, or both.

#### Outlook

Space organizations worldwide as well as private companies have declared their intent to build a permanent base on the Moon and from there, taking advantage of the lower gravity, to send humans to Mars. As such, lunar exploration has been thriving. As part of the commercial lunar payload service program (CLPS), two experiments have been selected to enable the first extraterrestrial magnetotelluric measurements in 2025 and 2027. Lunar Magnetotelluric Sounder (LMS) 1 successfully landed in Mare Crisium (mission results have not been announced at the time of writing), and LMS 2 is planned to land at Schroedinger crater, both with a setup that can launch electrodes out to a 20 m distance while carrying magnetometers. These measurements allow for more shallow sounding of the crust (Fuqua Haviland et al. 2022; Grimm and Delory 2012). Further experiments such as lunar magnetic observatories as part of a broader geophysical network (e.g., Weber et al. 2021; Kawamura et al. 2022) or instrumentation on rovers or carried by astronauts might soon be available.

Compared to Apollo-era studies, the use of higher precision instruments, long-duration time series, and advanced computational techniques offers a unique opportunity to revisit outstanding questions with significantly improved accuracy and resolution. Such questions include the presence of a partially molten basal layer (Grimm and Delory 2012) or possible compositional heterogeneity within the lunar interior, possibly suggesting incomplete magma ocean mixing and a complex thermal evolution.

### Mars

Mars has recently been the subject of extensive geophysical investigations due to the NASA InSight mission and particularly a highly sensitive seismometer deployed on the martian surface (Banerdt et al. 2020). Seismic measurements indicate a large core, implying a large fraction of light elements to reconcile the core’s size with the planet’s overall density (Stähler et al. 2021). Constraints on mantle structure have primarily been derived from seismic events originating from a tectonically active region north-east of the lander, Cerberus Fossae (Stähler et al. 2022; Khan et al. 2021). While seismic travel times from these events provide valuable information about Mars’ internal structure, they depend heavily on the correct identification and interpretation of seismic phase arrivals. Additional information from EM data would thus be a valuable independent contribution. Despite multiple missions with magnetometers on Mars, EM sounding has been shown to be particularly challenging on Mars (Mittelholz et al. 2023; Grimm et al. 2024).

#### The Magnetic Field Environment

Mars’ external magnetic field environment does not fully align with any of the cases in Fig. 6. While Mars currently has no active dynamo field (Acuña et al. 1999), strong crustal fields provide evidence for an ancient dynamo field with the youngest indications for an active field at 3.7 Ga (Mittelholz et al. 2020; Mitteholz and Johnson 2022). These locally concentrated crustal fields interact with the martian external magnetic field environment, including its ionosphere and the IMF, so that a Venus-like, but less symmetric, induced magnetosphere forms. Strong crustal fields concentrated in the southern hemisphere build so-called mini-magnetospheres which affect the degree of reconnection with IMF field lines and magnetic topology. In general, the crustal field has been modeled and can be subtracted from the data (Mitteholz and Johnson 2022), but fields from crustal field interactions with external fields remain (Brain et al. 2003; Mittelholz et al. 2017).

External field observations come from satellite measurements (MGS and MAVEN) and local surface observations (InSight and Zhurong). The MGS orbit is more suitable for observing periodic signatures, because altitude and local time are approximately fixed (Fig. 4a). The spatial coverage of MAVEN allows a more detailed representation of the entire magnetosphere (Ramstad et al. 2020; Gao et al. 2024), but also highlights its complexity and dynamic nature, capturing a wide range of spatial and temporal variations along the orbit.

Hence, MGS data give a detailed description of the magnetic field at 400 km and show that the dominant contribution at that altitude comes from the ionosphere (Brain et al. 2003; Mittelholz et al. 2017). As such, a diurnal signature is most dominant with strong magnetic fields at the day-side. This day-night pattern can also be observed from the ground with InSight (Johnson et al. 2020; Mittelholz et al. 2020). At shorter periods and around 100–1000s of seconds, pulsations have been observed in orbit (Glassmeier and Espley 2013; Dubinin and Fraenz 2016) and on the ground (Johnson et al. 2020; Mittelholz et al. 2023). The influence of the draped IMF at that altitude leads to a pattern that changes polarity at the Carrington rotation period of $\sim$26 days (Brain et al. 2006; Mittelholz et al. 2017, 2023).

Further, the effect of solar events has been observed from the surface and orbit. On the surface, such effects include increased peak-to-peak variations of the typical diurnal pattern and increased high frequency activity for up to 3 nights following relatively weak events (Mittelholz et al. 2021). In orbit, solar events of varying intensities have been observed and lead to sudden enhancements in the field when observed in the IMF and more complex ionospheric responses due to the dependence of local ionospheric conditions and crustal field interactions (Lee et al. 2017, 2018). The rich inducing field environment suggests that EM sounding on Mars is feasible and I will discuss attempts from orbital and surface data.

#### Conductivity Structure

**Fig. 11:**
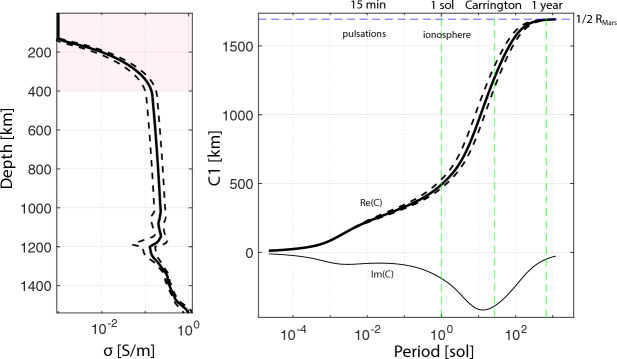
**a** Martian conductivity model derived from recent seismically constraint temperature profiles (Khan et al. 2023) in combination with a compositional model by Katsura et al. (2021) assuming high iron content in the mantle. Profiles show a mean, lower and upper bound from the range of temperature models, the pink shaded area highlights the lithosphere. Crustal conductivities from such models are only badly constrained and generally highly resistive and I set a lower bound of $10^{-3}$ S/m. **b** Resulting $C_1$ response from corresponding profiles in (**a**) using the recursive formula from Kuvshinov and Semenov (2012)

Informed by multiple missions and data sets acquired for Mars, electrical conductivity can be derived from compositional and temperature models. From these, one can forward model expected global *C*-responses (Fig. 11) for a given period range and under the assumption of a spherically symmetric (1D) conductivity structure. For large-scale inducing fields, the resulting *C*-response indicates that diurnal periods, particularly combined with diurnal harmonics, are suitable for mantle sounding. Longer period variations, such as those from Carrington rotations and annual variations could extend sensitivity deeper into the planet. This implies that current data sets should be sufficient to enable meaningful constraints on interior conductivity structure.

***Satellite data***

Among available datasets, the MGS mission offers the most suitable platform for electromagnetic sounding, primarily due to its regular orbit. In contrast, MAVEN samples different regions of the martian magnetic field environment within one orbit incorporating spatial and temporal variations. This variability complicates the accurate identification of induced field. However, in general, some aspects need to be considered: At daytime, electrical conductivity is large and effectively shields the planet. In addition, the ionospheric peak is generally below or at the lowest satellite altitudes, and the measured field during the daytime is likely not a potential field. However, at the nightside conductivity drops by approximately two orders of magnitude and a potential field approximation may hold. A study by Civet and Tarits (2014) has attempted to derive conductivity from MGS data. However, their approach, which relied on heavy data selection and lacked thorough discussion, raises concerns about the robustness of their conclusions. I refer to their manuscript for further information.

***Surface observations***

At the surface, InSight could be suitable to derive local transfer functions, and specifically diurnal periods and harmonics that stand out in the InSight data would seem promising. However, multiple challenges have been reported (Mittelholz et al. 2023, 2020; Grimm et al. 2024). Among those, contamination of InSight data is a severe limitation. Because the InSight Fluxgate Magnetometer was only an auxiliary instrument, designed to measure magnetic fields irrespective of their origin (this includes lander-generated fields), the associated cleanliness program was limited. As a result, data calibration efforts performed on the ground were imperfect and, especially at diurnal periods, the data are likely contaminated. This is because lander fields also vary at diurnal periods. A further approach of a combined analysis of MAVEN and InSight data failed because of lacking coherence between the field at the surface and in orbit. This might be the result of lander contamination, limited data covering MAVEN overflights, a too dynamic ionospheric environment of MAVEN (possibly violating a potential field description), or a combination of those factors.

#### Outlook

Past efforts to set up a network of geophysical stations, including magnetometers for the Netlander mission (that was unfortunately never realized), are well documented (Menvielle et al. 2000; Mocquet and Menvielle 2000). In the context of preparing for such a mission, Pinçon et al. (2000) discussed possibilities in using the horizontal gradient method to evaluate electrical conductivity from several surface magnetometers. At present, ESCAPADE is still expected to launch following the delivery of the spacecraft in 2024; however, the launch has been delayed, and no firm announcement has been made regarding a new launch date. Its precessing orbit may present challenges for EM sounding similar to MAVEN. However, the twin spacecraft configuration might facilitate novel sounding approaches. Beyond that, the future of Mars magnetic field exploration remains uncertain, with no magnetometry missions in development. Consequently, for the near future, existing datasets remain the primary resource despite unsuccessful attempts to date.

### Jupiter and the Galilean Moons

Hypotheses regarding subsurface oceans on outer solar system moons are supported by multiple lines of evidence from mission data, including indications of young, icy surfaces ranging from a few kilometers to tens of kilometers thick, overlying liquid water oceans (Marusiak et al. 2021). EM sounding provides a particularly powerful detection tool due to the highly conductive character of saline oceans (Seufert et al. 2011). As such, key evidence for these previously hypothesized oceans comes from magnetic field data from the Galileo spacecraft (Khurana et al. 1998; Neubauer 1998; Kivelson et al. 2000), underlining the importance of EM sounding in exploring planetary bodies. A renewed focus on those bodies is especially timely due to upcoming missions, such as JUICE and Europa Clipper, that will provide critical new datasets to further explore the presence and properties of these hypothesized oceans.

Europa’s distinctly characteristic surface shows cracks across an 80–170 km thick icy surface. Multiple lines of evidence support the idea that this ice layer overlies an ocean (Pappalardo et al. 1999). Callisto and Ganymede might also host a subsurface ocean, although their internal structures remain more uncertain and continue to be debated (Khurana et al. 1998). For the volcanically active Io, intense volcanic activity suggests the possible presence of a subsurface magma ocean, but the existence and exact nature are still investigated (Khurana et al. 2011). Spacecraft have sampled the Jovian system, but the resulting data are sparse, and studies so far rely on only a few flybys mostly by Galileo with additions from Juno (Fig. 5). I will first discuss the magnetic environment in which the Galilean moons are embedded, followed by a discussion of EM literature, first broadly and then individually summarizing the current understanding of each of the Galilean moons, in order of increasing distance from Jupiter.

#### Inducing Field

**Fig. 12:**
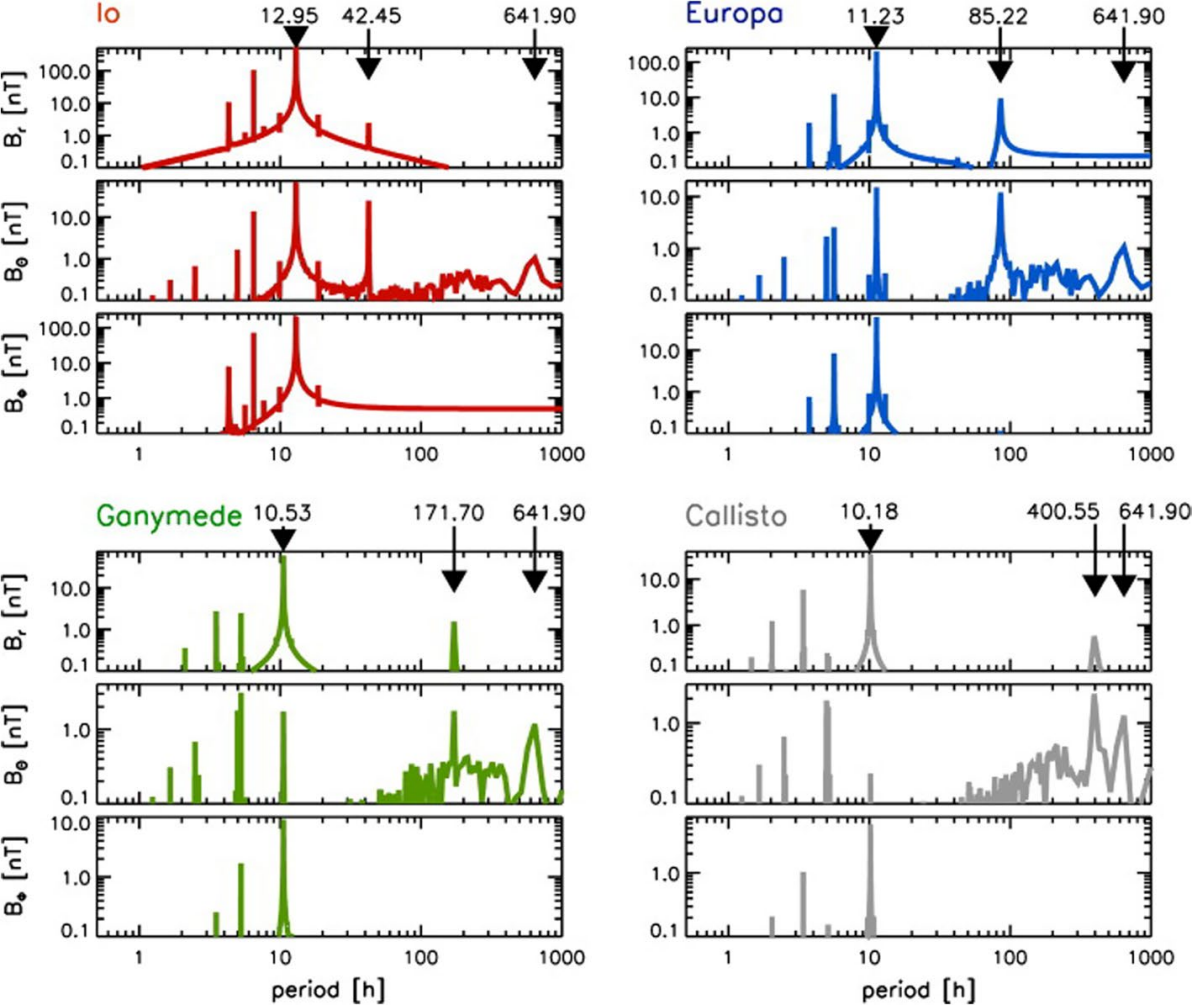
Figure from Seufert et al. (2011): total amplitude spectra for Io (red), Europa (blue), Ganymede (green) and Callisto (gray). Arrows indicate the synodic, the orbital and the solar rotation period, respectively. Note that the scale of the y-axis varies from panel to panel. Amplitudes and periods are tabulated in their Table 2

Galilean moons orbit within Jupiter’s strong magnetic field and the prime inducing field is due to the 9.6 degree tilt of Jupiter’s magnetic dipole moment with respect to its rotation axis. Because Jupiter with its field rotates faster than the moons' orbital period, their position fluctuates between above and below the Jovian magnetic equator. The Jovian synodic rotation period, defined as the time it takes for the respective moon to return to the same Jovian longitude, is 12.95, 11.23, 10.53 and 10.18 hours for Io, Europa, Ganymede, and Callisto, respectively. These periods, along with their harmonics, dominate the temporal variations of the inducing field (Fig. 12). Further, Jupiter’s field contains higher-order moments (Connerney et al. 1999; Khurana et al. 2004), and is distorted by currents in the plasma sheet that stretch the magnetospheric fields and increase the contribution of the radial component. This effect increases with distance from Jupiter (Neubauer 1998, 1999). Because Jupiter’s magnetospheric current sheet mostly co-rotates with the magnetic equator, this leads to additional field variations at the synodic rotation period.

The rotation period of the moons themselves is a further period of importance (42.45, 85.22, 171.70, and 400.55 hours for Io, Europa, Ganymede, and Callisto, respectively). Their inclination and eccentricity lead to changes in latitude and distance to Jupiter’s internal field. Also, as for all bodies discussed so far, the solar rotation period and harmonics are a source of inducing fields varying at a period of about 640 hours and its harmonics. Although additional sources of inducing fields exist (Seufert et al. 2011), electromagnetic sounding studies to date have primarily focused on the dominant inducing signal associated with the synodic rotation period (Fig. 12).

Finally, Seufert et al. (2011) evaluated the amplitudes of the inducing fields in addition to the spectrum shown in Fig. 12. The radial component at Jupiter’s rotational period reaches up to $\sim$750, 215, 85 and 40 nT for Io, Europa, Ganymede, and Callisto, decreasing with distance to Jupiter. In addition, Jupiter’s magnetospheric plasma interacts with the atmospheres and ionospheres of its moons, generating magnetic field perturbations that also vary depending on their position relative to Jupiter’s plasma sheet. At Europa, such interactions produce perturbations of approximately 10 nT when outside the plasma sheet and exceed 100 nT within it (Schilling et al. 2007). Ganymede experiences disturbances of around 50 nT outside the plasma sheet and up to 100 nT inside (Kivelson et al. 2002; Duling et al. 2014). At Callisto, plasma-induced fields range from a few nT to roughly 10 nT, depending on location relative to the plasma sheet (Zimmer et al. 2000; Liuzzo et al. 2018).

#### Detecting and Characterizing Oceans

For a spherically symmetric body, subjected to a uniform oscillating external magnetic field, the induced magnetic field forms a dipole that oscillates in response to the external field (Saur et al. 2010). The strength and phase shift of this induced field are influenced by the body’s radial conductivity profile and the frequency of the external oscillation, offering a method for investigating a body’s internal structure and, particularly, detecting their oceans. Hence, a spherical conducting shell model (Sect. 2.3.1), based on the work of Lahiri et al. (1939) and Srivastava (1966), then summarized in the context of planetary-scale problems (Parkinson 1983) was proposed by Zimmer et al. (2000); this study remains the basis for the literature in the field.

**Fig. 13:**
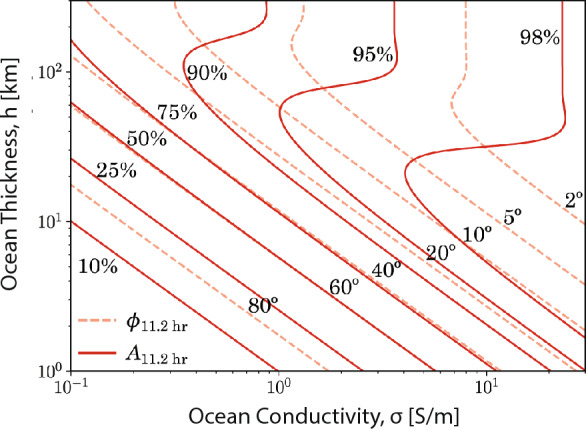
Figure from Biersteker et al. (2023) and after Zimmer et al. (2000) showing amplitude and phase lag of the inducing field in response to a primary magnetic field $B_p$ of amplitude 200 nT at the European synodic period (11.2 h) as a function of ocean conductivity, $\sigma$, and thickness, *h*. Assumed ice shell thickness is *d* = 0 km

Generally, a perfectly conducting ocean would induce the largest response, with an amplitude equal to the inducing field. In this case, the ocean would fully shield the interior from the inducing field. In reality, ocean conductivity is finite, and the induced response amplitude is smaller than the inducing field with a phase delay, depending non-uniquely on the subsurface structure. However, the layer model approach has allowed identifying high-conductivity subsurface regions that can best be explained by an ocean. The trade-off between ocean thickness and ocean conductivity for observed values of amplitude and phase, here for the synodic rotation period of Europa, is shown in Fig. 13. It is clear that degeneracies persist if the induction response can only be measured at a single frequency. When ocean thickness is less than $\sim$20 km and ocean conductivity is low, the contour lines for amplitudes and phase lag are parallel and one cannot uniquely determine both, ocean thickness and conductivity. In contrast, when the ocean thickness exceeds 40 km and the conductivity is higher than 1 S/m, the contours intersect. These intersections can reliably yield distinct values for ocean conductivity and thickness. Moreover, by observing different inducing periods, one can determine three ocean characteristics: burial depth, thickness, and conductivity (Kivelson et al. 2023). Thus, in addition to detection and parameter trade-off studies, recent research has explored the inherent assumptions of existing layered models, as well as the potential for multi-frequency studies using upcoming data sets.

For example, Styczinski and Harnett (2021) highlighted the inadequacy of assuming a spherical geometry for ocean worlds. The unique characteristics of these moons, such as their small size, low density, and proximity to their parent planets, result in significant gravitational asymmetries, which are further influenced by features like tidal heating and inhomogeneous ice shells, as supported by observations suggesting present-day cryovolcanism and diapirs (see, e.g., Rathbun et al. (1998); Pappalardo and Barr (2004)). To address these complexities, Styczinski et al. (2022) expanded the outer boundary using spherical harmonics. By assuming a body with a globally conducting layer exposed to a uniformly oscillating field, they demonstrated that each spherical harmonic in the shape expansion independently induces a discrete magnetic moment, allowing the total induced magnetic field to be calculated through superposition. This approach enables modeling the effects of tidally deformed oceans.

Moreover, Vance and Goodman (2009) and Vance et al. (2021) examined the impact of salinity and nonuniform electrical conductivity in oceans, influenced by factors such as adiabatic temperature gradients and stratification. Vance et al. (2021) investigated model responses, accounting for the depth-dependent electrical conductivity of the oceans due to varying temperature and pressure, while also incorporating the effects of motionally induced magnetic fields generated by internal ocean currents. They estimated a maximum amplitude of 20 nT for the radial ocean induced magnetic field component, i.e., a large contribution to the overall signal (e.g., for Europa the driving field amplitudes for the synodic and at orbital period are 250 nT and 14 nT). However, in a more recent study that focused on Europa’s motionally ocean-induced magnetic field, amplitudes of less than 1 nT were proposed (Šachl et al. 2025). The difference was mainly attributed to the comprehensive solution of the electromagnetic induction equation (Šachl et al. 2025), as opposed to the more simplified scaling used by Vance et al. (2021) in combination with slower and more realistic values of ocean current velocities.

Additionally, Biersteker et al. (2023) developed a framework for characterizing the interior structure of these moons using multi-frequency induction and Bayesian inference. Their work focused on solving for ocean conductivity, ocean thickness, and ice shell thickness. They demonstrated that accurate retrieval of the ice shell thickness is highly dependent on precise modeling of magnetic field interactions with ambient plasma, whereas ocean thickness and conductivity are less sensitive to such parameters. They also described how constraints from gravity or radar-derived ice shell thickness could improve their results.

Furthermore, an intriguing consequence of the interaction between the moon’s atmospheres and the Jovian energetic environment is the formation of auroras. Far-ultraviolet (FUV) observations from the Hubble Space Telescope have captured dynamic auroral ovals on these moons (Saur et al. 2010). The Jovian magnetic field is expected to induce an oscillatory pattern in these auroral ovals, causing them to shift about the plane perpendicular to the magnetic field. However, the degree of these oscillations is reduced when a strongly conductive layer is present beneath the surface (Saur et al. 2015; Roth et al. 2017). This phenomenon has been utilized to compare observed auroral oscillations with theoretical predictions, providing independent evidence for or against subsurface oceans on Ganymede (Saur et al. 2015) and Io (Roth et al. 2017).

#### Io

Io is the most volcanically active body in our solar system, presenting volcano-tectonic features, hot spots, and plumes. The main source of internal heating comes from tidal dissipation due to Io’s eccentric orbit (Peale et al. 1979). In the context of Io, the presence of a magma ocean has thus been discussed widely and differentiates this body from the other Galilean moons.

Khurana et al. (2011) first hypothesized that Io’s magnetic field data support the presence of a global conducting layer, arguing that the response of a solid mantle cannot account for observations. They concluded that an approximately 50 km magma ocean with a melt fraction of tens of percent is present below a low density crust of 30–50 km thickness.

However, following this study, Roth et al. (2017) studied auroral spots caused by interactions between Io’s atmosphere and Jupiter’s magnetosphere. Observed oscillations in phase with Jupiter’s time-varying fields showed a reduced amplitude compared with the magnetic field oscillations, consistent with an expected induction response. However, Roth et al. (2017) also suggested that a magma ocean, that is, a highly conductive layer near the surface, would be expected to lead to a phase shift that is not observed in the data. This put an upper bound on the conductance of the near-surface layer to $1 \times 10^3 S$ and suggested the conductive core as a possible driver of the in-phase oscillations.

Further, heterogeneity in Io’s atmosphere was shown to lead to significant perturbations of plasma and magnetic field observations (Blöcker et al. 2018). As such, magnetic field perturbations from Galileo flybys can be entirely explained by the interaction of plasma with the asymmetric atmosphere, in which case no magma ocean is required.

Miyazaki and Stevenson (2022) explored a solid layer with a high melt fraction (porosity > 20 %), termed ’magmatic sponge’, and found it to be unstable, rapidly separating into two phases and forming a subsurface magma ocean. This ocean, while likely containing crystals, would behave like a liquid. This idea is consistent with conclusions from Roth et al. (2017) and Blöcker et al. (2018), as the thin (1–10 km) ocean would produce only a weak magnetic signal. Juno observations recently further supported this, showing that the heat distribution of Io aligns with the presence of a global magma ocean or, alternatively, shallow internal heating (Davies et al. 2024).

#### Europa

As mentioned, multiple data sets corroborate the existence of a European ocean. Several Galileo flybys showed clear dipole contributions consistent with a subsurface ocean induced by a time-varying field at Jupiter’s synodic rotation period (Khurana et al. 1998; Kievelson et al. 1999; Kivelson et al. 2000). While the initial observation came from a single flyby (Kievelson et al. 1999), further flybys confirmed the ocean hypothesis (Kivelson et al. 2000). Using the conductive spherical shell approach (Sect. 2.3.1), Zimmer et al. (2000) then proposed a lower limit for the inductive response amplitude of 0.7 and constrained the depth of the ocean layer to less than 200 km with a conductivity of at least 0.06 S/m. Further investigations by Schilling et al. (2004) examined the presence of a fixed dipole field within Europa and established an upper limit of 25 nT, significantly smaller than the several 100 nT amplitude associated with the induced field, confirming the presence of an inductive response.

Subsequent investigations included more complex modeling that incorporated Europa’s ionosphere (Kliore et al. 1997), which interacts with Jupiter’s magnetosphere to generate substantial current systems (Saur et al. 1998; Saur 2004). A self-consistent induction model including a three-layer shell, comprising a core, mantle, and subsurface ocean, surrounded by an insulating crust and plasma currents, provided better constraints on Europa’s interior (Schilling et al. 2007, 2008). This model supported ocean conductivities of at least 0.25 S/m of 100 km thickness. These results are consistent with the presence of a liquid saline ocean, effectively ruling out scenarios involving either a frozen or soft ice layer.

While all previous work relied on the synodic rotation period, Seufert et al. (2011) performed a comprehensive analysis across a range of periodicities using a magnetospheric field model that included Jupiter’s internal field, a current sheet field, and contributions from magnetopause boundary currents. Induced magnetic signals were calculated for various multi-layer models and oceans with varying conductivity. The study demonstrated that subsurface oceans could be well detected at several periods, even when relatively thin and only moderately conductive. It also showed that effects of mantle induction are negligible for mantle conductivities up to $10^{-3}$ S/m, and that signals from an interior core are very small and likely undetectable. Given the non-linear dependence of signal amplitudes on interior structure, the study emphasizes the importance of multi-frequency analysis for reliably constraining both ocean thickness and conductivity (also see Fig. 13 and the discussion in the previous section).

To cover a wider parameter space, Biersteker et al. (2023) applied their Bayesian framework to synthetic Europa Clipper spacecraft data at variable frequencies using a three-layer internal structure model of varying parameters. In most cases, they were able to successfully recover ocean conductivity and ice shell thickness, while ocean thickness was more dependent on the internal structure model and in some cases they could not provide a robust estimate without additional information. The incorporation of gravity and radar data, both part of Europa Clipper’s payload, was highlighted as a promising pathway to resolve these ambiguities and improve the characterization of Europa’s subsurface ocean.

Lastly, studies investigating ocean asymmetries were conducted on a global (Styczinski and Harnett 2021; Styczinski et al. 2022) and local (Winkenstern and Saur 2023) scale. Globally, and specifically for Europa, the effect of a tidally deformed ocean accounts for a difference in the induced magnetic field of more than 2 nT near the surface. Locally, water reservoirs between a global spherical ocean and the surface introduce nonlinear coupling. Such effects are not detectable at 25 km altitude, i.e., future Europa Clipper orbital altitudes, but would affect surface measurements for reservoirs larger than 8 km for a conductivity of $\sigma$=30 S/m or 20 km for $\sigma$=5 S/m (Winkenstern and Saur 2023). Similarly, motional induction created by ocean currents will likely lead to signatures of $\sim$1 nT, although these depend strongly on the poorly constrained flow velocity (Šachl et al. 2025).

#### Callisto

Galileo flybys ruled out the existence of a fixed internal dipole field, but led to the hypothesis of a subsurface ocean on Callisto (Khurana et al. 1998; Neubauer 1998; Kievelson et al. 1999; Zimmer et al. 2000). While for Europa this finding was supported by geological observations, for Callisto, a body only partially differentiated, geologically inactive, and heavily cratered, this finding was more surprising. Early comparisons of Galileo magnetic data with single shell induction models (Zimmer et al. 2000) showed that conductivity needed to exceed 0.02 S/m at depths shallower than 300 km to explain observed signals. In these models, a larger ocean depth than that for Europa was permissible, possibly explaining the lack of geological surface indications of an ocean. However, the discovery of a substantial ionosphere around Callisto (Kliore et al. 2002) raises the question of whether induction within the ionosphere itself could lead to the observed signal. Ionospheric Pedersen and Hall conductivities are inversely proportional to the local magnetic field strength, as stronger magnetic fields restrict ion and electron mobility. At Callisto, the ambient field is weakest among all Galilean moons because of the greater distance to Jupiter and the lack of an internal field, and thus induction within the ionosphere could plausibly account for part of the observed signal. As a result, it has been debated whether the observed induced field is attributable solely to the ionosphere, an ocean, or a combination of both.

A recent model of Callisto’s ionosphere indicated that the ionosphere is likely spatially inhomogeneous and possibly transient (Hartkorn et al. 2017). Using this model, Hartkorn and Saur (2017) reinvestigated Galileo flybys and specifically if the “induction signal” could arise from an ionosphere instead. To address this, the authors built an analytical model to understand the effect of induction in the ionosphere. They further numerically derived the expected effect from induction owing to the interaction of Jovian time-varying fields and the ionosphere. Interestingly, they showed that the effect of induction within the ionosphere is comparable to the effect of induction due to an ocean (Hartkorn and Saur 2017). This is in contrast to Europa or Ganymede, where induction within the ionosphere leads to a signal of only $\sim$1 nT and $\sim$0.05 nT, respectively (Kivelson et al. 2023).

More recently, Cochrane et al. (2025) revisited this problem using a combination of inverse and ensemble forward modeling, coupled with previously published plasma interaction simulations. For the first time, all Galileo flybys were considered to constrain the external driving field, and three flybys (C03, C09, and C10) were used to assess the induction response. The study jointly considered the contributions from both a subsurface ocean and the ionosphere, concluding that the observed magnetic signal is best explained by the presence of a thick, conductive ocean in combination with a conductive ionosphere. Their results provided tighter constraints on Callisto’s internal structure, with best-fit models favoring thick ice shells and ocean conductivities in the range of 0.5 to 10 S/m.

Despite these advances, uncertainties associated with factors such as the nightside ionosphere and the specifics of plasma interactions continue to limit our ability to definitively confirm the presence of an ocean. Looking ahead, Liuzzo et al. (2018) evaluated the potential of the upcoming JUICE mission to resolve this ambiguity. Analyzing 12 planned flybys, they found that four (11C3, 14C6, 17C9, and 23C12) would occur close to the center of Jupiter’s magnetospheric current sheet, where plasma disturbances are expected to obscure any induction response. The remaining eight flybys, situated farther from the current sheet center, may offer more favorable conditions to detect magnetic signatures indicative of subsurface conductivity. However, the complex and possibly variable structure of Callisto’s ionosphere, not fully represented in their models, may still introduce significant challenges. Detailed magnetic analysis and careful interpretation will be required, particularly during low-altitude encounters.

#### Ganymede

Ganymede’s internally generated dipole field was detected and characterized using initially two, later six passes of the Galileo spacecraft, revealing an equatorial magnetic field strength of $\sim$720 nT, offset by 170$^{\circ }$ from the rotation axis (Kivelson et al. 1996, 1997, 2002). Of these, three flybys were consistent with the presence of a highly conductive layer in the first few hundred kilometers below the surface and the possibility of a subsurface ocean (Kivelson et al. 2002). However, a dipole field in addition to quadruple moments was also consistent with the data, and the few flybys were unable to distinguish between such spatially and temporally varying fields (due to induction). Later attempts to distinguish between quadrupole and induction models with additional data were also unsuccessful, and showed that the existing data do not allow us to uniquely answer if Ganymede has a subsurface ocean (Weber et al. 2022; Plattner et al. 2023).

Recently, Jia et al. (2025) presented an improved model of Ganymede’s magnetic field, using data from the Galileo and Juno spacecraft. The authors addressed limitations in previous models by incorporating magnetohydrodynamic (MHD) simulations to quantify the magnetic field perturbations caused by plasma and ionospheric currents. By removing these perturbations, they refined the estimates of Ganymede’s internal magnetic sources, considering both permanent and induced dipole models. Comparing the improved model with the data, they could still not distinguish between the quadrupole vs. induction hypothesis. However, they found a lower induction efficiency than previously proposed, which could indicate a deeper conducting layer (<150 km) or lower ocean conductivity (<1 S/m). In addition, specifically the Chapman-Ferraro magnetic field has been shown to also affect the induction signal (Kaweeyanun and Masters 2024). With an amplitude of approximately 50 nT it presents a significant contribution to the total field. These improvements in models are an important contribution, as fitting them to the data generally determines whether induction could lead to observed signals. Even after accounting for complexities related to the plasma environment, this has not been successful (Jia et al. 2025; Kaweeyanun and Masters 2024). Therefore, to truly untangle the signals that vary temporally and spatially, additional data is needed. Even just the first three JUICE flybys will in addition with Galileo data provide enough information to differentiate between induction and quadrupole signals (Sharan et al. 2025). Later, dense coverage will allow for detailed characterization of internal and external contributions, including ocean properties.

Complementary to above studies, the interpretation of auroras provided additional support for the existence of a conductive layer. These observations were consistent with conductivities equal to or greater than 0.5 S/m and at least 0.09 S/m for an ocean between 150 to 250 km depth (Saur et al. 2015). Lastly, laboratory measurements of aqueous $MgSO_4$ have been performed at high pressures (200–1200 MPa) and low temperatures (243–295 K) to provide information on electrical conductivity under Ganymede conditions (Pan et al. 2020). The resulting conductivity values were lower than previously predicted (Vance et al. 2018), converging with recent results of Jia et al. (2025).

#### Outlook

EM sounding has immense potential for advancing our understanding of the Jovian system, as it enables orbital exploration of subsurface structures across the diverse Galilean moons. The studies discussed so far are based on very little data in addition to modeling work, waiting to be verified by upcoming missions. Europa Clipper and JUICE, will provide critical new observations (Fig. 5), particularly for Ganymede and Europa, and will enable multi-frequency EM sounding alongside further complementary science investigations (Pappalardo et al. 2024; Kivelson et al. 2023; Van Hoolst et al. 2024). Building on the insights gained from studies of Europa, Ganymede, Callisto, and Io, future EM investigations promise to illuminate broader questions regarding planetary habitability, internal processes, and surface-ocean interactions.

### The Saturn System

The Saturn system hosts several moons that are considered potential ocean worlds with significant implications for astrobiology (Nimmo and Pappalardo 2016; Marusiak et al. 2021; Ermakov et al. 2021). The most prominent candidates include Enceladus, Titan (Fig. 2), but also Mimas, Dione, Tethys or Rhea.

#### Background

Enceladus is widely regarded as one of the most promising ocean worlds in the Saturn system. Observations from the Cassini spacecraft revealed the presence of plumes of water vapor, ice particles, and organic molecules erupting from the moon’s south pole, along the so-called “tiger-stripes” (Dougherty et al. 2006; Ermakov et al. 2021). These geysers are believed to originate from a subsurface ocean beneath Enceladus’ icy crust. The ocean, likely salty and in contact with the moon’s rocky core, may contain hydrothermal vents, providing a potential habitat for life. The interaction between the ocean and the core could supply the necessary chemical ingredients and energy sources to support microbial life Ermakov et al. (2021).

Titan, Saturn’s largest moon, also presents strong evidence for a subsurface ocean (e.g., Sohl et al. 2003). While the surface of Titan is dominated by lakes and seas of liquid methane and ethane, the data suggest the existence of a global ocean of liquid water mixed with ammonia beneath its thick ice crust. This ocean, located approximately 50 to 100 kms below the surface, could potentially harbor life, particularly if it contains essential chemical compounds and energy sources.

Recent studies suggest that Mimas, a smaller moon of Saturn, might also possess a subsurface ocean (Bradák and Okumi 2024, and references therein). The observed libration in Mimas’ orbit hints at the possibility of a partially fluid interior. However, this remains a topic of debate as confirming the presence of an ocean beneath what is likely a very thick ice shell is challenging. Dione and Tethys are also considered potential ocean worlds, although the evidence is less conclusive compared to Enceladus and Titan (Hussmann et al. 2006; Nimmo and Pappalardo 2016). Observations of their surfaces, combined with gravity data, suggest the potential for liquid water beneath their icy crusts. These subsurface oceans, if present, would add to the growing list of intriguing environments within the Saturn system that warrant further exploration.

#### The Magnetic Field Environment

One of the big surprises associated with Saturn’s internal magnetic field was the finding of its almost perfectly axisymmetric magnetic field, initially observed by Pinoeer 11 and Voyager flybys (Acuña et al. 1980; Ness et al. 1982), and later confirmed by the Cassini mission. The tilt of Saturn’s dipole axis relative to its rotation axis is less than 0.007$^{\circ }$ (Cao et al. 2020; Dougherty et al. 2018). According to Cowling’s theorem, an axisymmetric field cannot be sustained by a dynamo (Cowling 1933) and this finding thus challenged the fundamental understanding of MHD dynamo processes. Differential rotation in a stably stratified layer surrounding the dynamo region has been shown to “symmetrize” the field, i.e., dampen any non-axisymmetric components and is an explanation for these observations (Yan and Stanley 2021; Stanley and Bloxham 2016; Christensen and Wicht 2008).

Due to the axisymmetry, one would expect a lack of a tilt-driven inducing magnetic field at the synodic rotation period of 11 hours. However, so-called planetary period oscillations (PPOs) at the approximate synodic rotation period still exist, but are of external origin (Andrews et al. 2012; Southwood and Cowley 2014; Provan et al. 2019). The primary inducing field acting on Saturn’s moons is due to their eccentric orbit, and compared with the Jovian system, the inducing field strengths are small reaching only approximately 10 nT out to the orbit of Rhea. This is comparable in amplitude to the magnetic perturbation caused by Enceladus’ plume activity (Dougherty et al. 2006).

For satellites in the Saturninan system, no internally generated field has been observed. Also, no substantial atmospheres have been detected, with the exception of Titan and Enceladus, and their magnetic field environments are mostly Moon-like, i.e., resemble that of a non-magnetized body. A review by Simon et al. (2015) describes the interaction of some of the major Saturnian satellites with their plasma environments, and I refer to their work for this discussion. In the context of induction studies, it is important to note that the inducing fields out to Rhea are comparably small, and outside of Titan’s orbit, field variations are largely aperiodic.

#### Induction Studies

In the Saturnian system, the evidence for oceans comes mainly from geodetic investigations (Nimmo and Pappalardo 2016; Lunine 2017; Ermakov et al. 2021). Due to the small inducing fields, the separation of inducing and induced fields is difficult, and long-term observations that would allow data stacking are not available. Thus, the currently available flyby’s have not allowed deducing induction signatures for any of the moons.

Focusing on Enceladus, Saur et al. (2024) discussed its inducing field environment in more detail. The primary inducing field stems from its (small) orbital eccentricity (*e* = 0.005) that causes variations in Saturn’s magnetic field at Enceladus’ orbit. The latitudinal magnetic field varies by approximately ± 5.0 nT, while the radial component shows negligible variability ($\sim \pm$0.2 nT) due to higher order terms (Dougherty et al. 2018), and the azimuthal field remains zero due to the high axisymmetry. The sidereal orbital period, slightly modified due to precession of the periapsis, is $\sim$1.4 days. Previously, Saur et al. (2010) established that a global ocean at Enceladus could theoretically be detected using the orbital period, whereas a south polar pond would require shorter periods.

More recently, with a combination of interior conductivity models and Cassini-derived inducing magnetic fields, induced responses were derived (Saur et al. 2024). Close flybys of Enceladus lead to small magnetic field perturbations (1–3 nT) within larger plume-dominated variations (10–30 nT), potentially indicative of induction. In contrast, observed magnetic field perturbations during north polar flybys cannot be fully explained by MHD plasma effects alone, pointing to induced magnetic fields from Enceladus as the likely missing contribution. These findings could suggest that Enceladus porous core is highly conductive (20–30 S/m), as the ocean conductivity inferred from plume salinity measurements (1–3 S/m) is insufficient to account for the required induction response. Alternatively, ocean salinity and conductivity may be higher than currently estimated. However, an investigation of non-MHD plasma effects will have to be conducted.

For Titan, assuming an amplitude factor of 0.8 and using the fourth harmonic of the orbital period, an ocean conductivity of 9 S/m was required (Saur et al. 2010), again indicating the need for high conductivities. To conclude, while in theory measurements of induction might be possible, the Saturnian system is less suited for EM induction studies than the Jovian system or the ice giants as will be discussed in the next section.

#### Outlook

The Saturnian system and its many potential ocean worlds are the focus of current and future investigations. NASA’s Dragonfly mission, planned to investigate Titan in the 2030s, carries a geophysical package, which is, however, not equipped with a magnetometer (Barnes et al. 2021). Enceladus Orbilander mission concept has been endorsed as the secondary flagship mission in the last NASA decadal survey (National Academies of Sciences 2023), but, if implemented, it would only arrive in the 2050s (MacKenzie et al. 2021). ESA has proclaimed Enceladus as a top target for possible missions, but there are no concrete plans at the time of writing. Thus, the lack of upcoming possibilities for magnetic field investigations likely means that the near-term prospects for EM studies in the Saturnian system are limited.

### Icy Planets

Only a single Voyager flyby has collected data at Uranus and Neptune at distances of 4.2 $R_U$ and 1.8 $R_N$, respectively, including measurements of their magnetic fields (Ness et al. 1986, 1989; Hofstadter et al. 2019). Generally, those data show geologically active systems and unique magnetic field environments. One major difference between the systems is that the Neptune system only harbors one major moon, Triton, which is likely a captured Kuiper Belt object (Agnor and Hamilton 2006). Uranus on the other hand has multiple moons and the five innermost ones from closest ($\sim$5.1 Uranus radii) to farthest ($\sim$22.8 Uranus radii) are Miranda, Ariel, Umbriel, Titania, and Oberon. Thermal modeling indicates that Titania and Oberon at Uranus, as well as Triton at Neptune, could have recent subsurface oceans with heat supplied by tidal heating, gravitational energy and radiogenic elements (Nimmo and Pappalardo 2016; Hussmann et al. 2015).

Uranus’ moons, particularly Ariel and Miranda, show evidence for rifting, faulting, and extension potentially above buoyant diapIrs, indicating a rich geologically active history that has not been explored in detail beyond Voyager II (Schenk and Moore 2020; Pappalardo et al. 1997; Beddingfield and Cartwright 2020; Croft and Soderblom 1991; Smith et al. 1986). Ariel and Miranda have been suggested to harbor subsurface oceans because landforms are suggestive of recent cryovolcanism and their surfaces might be as young as 0.1 billion years (Schenk 1991; Zahnle et al. 2003). On Ariel’s surface, ammonia-bearing species have been identified in spectroscopic observations, and because charged particles would destroy those on relatively short time scales; these observations suggest recent emplacement, possibly connected to a recent subsurface ocean (Cartwright et al. 2020).

Triton orbits Neptune in a retrograde manner and is thus thought to be a captured moon. It has a tenuous nitrogen atmosphere, and Voyager 2 detected geyser-like vapor plumes (Smith et al. 1989) that could originate from a global ocean, sustained by tidal and radiogenic heating (Nimmo and Pappalardo 2016).

#### Uranus’ Magnetic Field Environment

Voyager’s magnetic field measurements at Uranus revealed a global field tilted at an angle of 60$^{\circ }$ from its rotational axis, significantly offset (0.3 $R_\textrm{Uranus}$) from the planet’s center, where the rotational axis itself is tilted by 97$^{\circ }$ from the orbital plane around the Sun. The internal field, thought to be generated in the convective and electrically conductive ice-rich mantle layer, is not dipole dominated (Stanley and Bloxham 2004, 2006). The dipole moment is approximately 50 times that of Earth’s (3.9 $\times$ 10$^{17}$ Tm$^3$) (Russell and Dougherty 2010), and due to the field’s complex structure, the surface magnetic field strength varies significantly, from roughly 10,000 nT to 100,000 nT.

These characteristics have important implications for the magnetic field environment experienced by Uranus’ moons. Due to the extreme tilt of the magnetic field axis, the moons encounter changing magnetic field environments during a synodic period. Inducing field amplitudes vary from approximately 4 nT at Oberon to 330 nT at Miranda, depending on the moon’s distance from Uranus and the geometry of the planetary field. These synodic period variations are generally identified as the dominant inducing fields. The moons themselves follow nearly circular orbits (eccentricities 0.0011 to 0.0039) and mostly lie within Uranus’ orbital plane. Miranda is an exception with an orbital inclination of 4.2$^{\circ }$. Thus, magnetic induction effects at moon’s orbital periods are considered secondary compared to synodic variations.

Also, the magnetic field spatial offset with respect to the Uranus’ center is particularly relevant for the closer satellites Miranda and potentially Ariel (Arridge and Eggington 2021). Additionally, the subsolar magnetopause distance, located at approximately 19 $R_U$, lies within the orbital range of Titania (17.1 $R_U$) and Oberon (22.8 $R_U$). As a result, they can cross the magnetopause boundary and experience transitions between magnetospheric and solar wind environments depending on orbital phase, season, and solar wind activity.

**Fig. 14:**
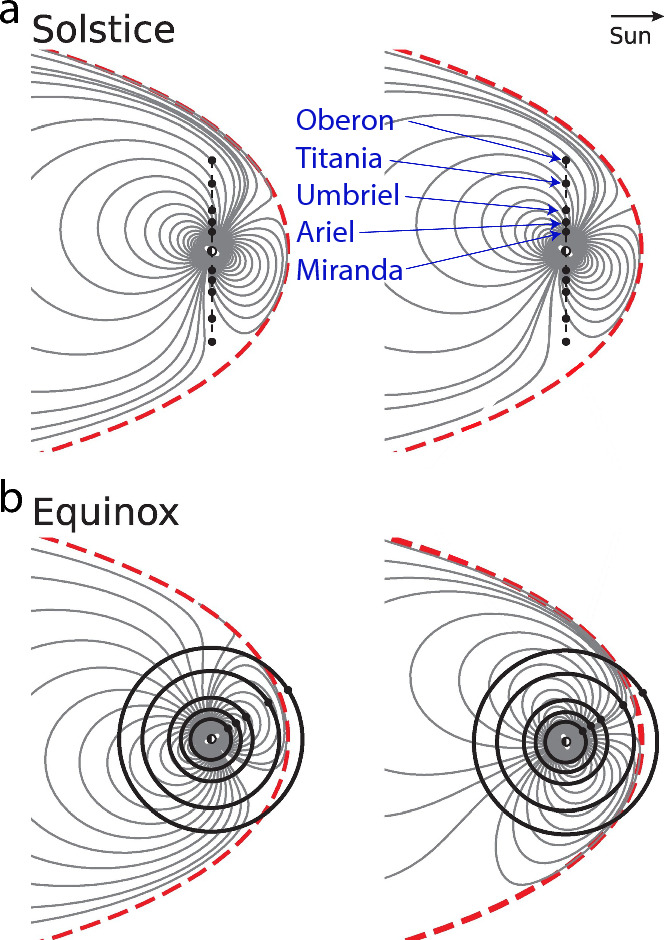
A schematic of Uranus’ magnetosphere modified from Arridge and Eggington (2021) showing field lines (gray) and the magnetopause boundary (red dashed) at **a** solstice and **b** equinox, for two different rotational phases each. The moons are shown (black dots) with their orbital planes (black lines)

Lastly, Uranus’ extreme obliquity leads to strong seasonal effects. Given that a Uranian year lasts 84 Earth years, the magnetic field environment at the moons’ locations can vary substantially depending on the timing of potential missions. Accurate modeling of the inducing field thus requires consideration of the seasonal phase at the time of observation or spacecraft arrival (Fig. 14).

#### Neptune’s Magnetic Field

Neptune’s magnetic field is tilted at an angle of about 47$^{\circ }$ from its rotational axis and has a more moderate obliquity of 30$^{\circ }$ as opposed to Uranus. Like Uranus’ field, it is not dipole dominated (Stanley and Bloxham 2004, 2006) and also significantly offset from the planet’s center. The magnetic moment is about $2.2\times 10^{17} Tm^3$ and 25 times stronger than that of Earth (Russell and Dougherty 2010). The combination of Neptune’s magnetic field tilt and Triton’s orbital obliquity leads to strongly varying magnetic fields at Triton, which orbits at 14.4 $R_\textrm{Neptune}$ and is thus always within the Neptune magnetosphere (Ness et al. 1989). Dominant periodicities include Neptune’s synodic rotation period of 14 hours and Triton’s orbital period of 141 hours, and associated magnetic field fluctuations are between 3 and 12 nT (Saur et al. 2010).

#### Induction Studies

Due to the sparsity of data, induction studies so far have explored the model response to the inducing field of Uranus for different moons (Weiss et al. 2021; Arridge and Eggington 2021; Cochrane et al. 2021). Their assessment generally focused on the question of whether an ocean could be detected from a single flyby. All mentioned studies used a 3 layer model as described in Sect. 2.3.1 and discussed slightly different aspects of the induction response of different moons and the effect of varying ocean thickness, surface ice layer thickness, and electrical conductivities.

To model the inducing field, an internal magnetic field model was used; the model “AH5” up to the maximum spherical harmonic degree 4 (hexapole) was derived from Voyager 2 magnetometer flyby data and UV spectrometer aurora observations (Herbert 2009). Because of the single flyby, auroras at the magnetic field line footprints serve as additional constraints used to determine the higher multipole moments of the sparsely sampled global magnetic field.

Arridge and Eggington (2021) also incorporated a geometrical model for the magnetopause, as they included the outer moons and a full Uranian year in their simulation. Weiss et al. (2021) considered all five inner moons, but focus on Ariel. Their model neglected the presence of a possible ionosphere and magnetospheric current systems. In contrast, Cochrane et al. (2021) focused on inner moons Miranda, Ariel, and Umbriel and explored the potential to distinguish between induction signatures from subsurface oceans, with varying degrees of conductivity and depth, and an ionosphere.

All of these studies showed a rich spectrum of inducing fields, with strong signals at the inner moons, and considerable seasonal dependence for the outer moons. In addition, all studies concluded that ocean detection at the location of the moons is theoretically possible. However, Arridge and Eggington (2021) noted the much smaller amplitude of the induced response compared to Europa due to the thick inferred overlying ice shells for Uranus’ moons (Hussmann et al. 2006). Generally, the innermost moons Miranda, Ariel and Umbriel are exposed to particularly strong variations of the Uranus fields and in almost all model scenarios an ocean could be detected in a single flyby (Cochrane et al. 2021; Weiss et al. 2021). Even with a highly conductive ionosphere and realistic levels of instrument noise, the detection of an ocean with an electrical conductivity of 2 S/m and thickness of > 10 km was possible (Cochrane et al. 2021). The degree to which ocean thickness and conductivity can be constrained was dependent on interior characteristics and was, unsurprisingly, more favorable for thick and conductive oceans. Weiss et al. (2021) concluded that for an ocean conductivity of > 1 S/m and an ocean thickness of > 20 km, one can determine the thickness of ocean and ice, as well as ocean conductivity, with multiple flybys. Further out, a model flyby at 200 km altitude above the surface of Titania could provide clear identification of a thick ocean, but limited detectability for a thin ocean of 16 km (Arridge and Eggington 2021). For the case of Oberon, seasonality needs to be taken into account due to its proximity to the magnetopause, an important consideration for future missions.

Neptune’s moons have received less attention, and the only induction studies were focused on Triton, Neptune’s largest moon. Liuzzo et al. (2021) used a hybrid model that simulates plasma interactions with Triton’s ionosphere and a hypothetical induced field, considering the varying orientations of Neptune’s magnetic field relative to plasma flow. They found that magnetic field fluctuations and plasma characteristics in Triton’s magnetosphere and ionosphere remained stable despite changes in the surrounding environment. Features of Triton’s induced magnetic field were found to be visible near the surface under different magnetospheric conditions, plasma interactions, and inductive responses. In addition, a recent paper proposed a method for detecting a subsurface ocean on Triton, using data from a single flyby (Khurana et al. 2024). They focused on two periods, the synodic rotation period (14.4 hours) and the Triton orbital period due to Triton’s large inclination (141 hours). With an height-integrated ionospheric conductivity of $10^4$ S/m, the synodic period was shown to create a large response that would obscure any ocean induction response. However, a period of 141 hours would allow a successful ocean detection.

#### Outlook

The Neptune system, and specifically Triton, was highlighted as a target of interest for future exploration. The Trident mission concept (Frazier et al. 2020) aimed to characterize the moon in a single flyby and within the budget of a NASA Discovery mission; however, the mission was not selected. Based on discussed studies and the multiple candidate ocean worlds, Uranus might be the more promising system for EM sounding studies, and NASA plans align with that. The recent NASA decadal survey recognizes that the outermost Solar System is understudied and recommends a mission to Uranus as an upcoming flagship mission, with priority targets Ariel and Miranda (National Academies of Sciences 2023). This aligns with the broader objective of investigating Ocean Worlds and assessing potentially habitable environments beyond Earth, in parallel with upcoming missions to the Jovian system (Sect. 3.5). If launched in 2030, a Uranus mission would reach its destination in the mid-2040s. As such, data from this mission will not be available in the near future. However, modeling studies remain essential for refining scientific objectives, guiding instrument design, and informing mission concept development.

### Everything In-Between

Beyond the planetary systems in our Solar System, we must not overlook the space in between and beyond. Grayver (2024) included a quick summary of exoplanet research in his EM review, and I refer to that and references therein. In-between, I will only highlight two bodies, Ceres and Psyche, a dwarf planet and an asteroid, both in the main asteroid belt between Mars and Jupiter.

Ceres has been explored by the Dawn mission and is the most water-rich world (by mass percentage) in the inner solar system. Multiple lines of evidence including observations of recent or ongoing geologic activity (De Sanctis et al. 2020) and the inference of water below an ice-rich crust (Raymond et al. 2020; Fu et al. 2017), points toward Ceres being an ocean world (Castillo-Rogez 2020). As such, it represents a body in a magnetic environment similar to that of the Moon but much more conductive. Using a hybrid plasma model, Poppe and Fatemi (2023) examined how the solar wind interacts with Ceres under various internal conductivity structures (including a crust, mantle, and/or subsurface ocean) and different solar wind and IMF conditions. The results indicated that the interaction of the solar wind with Ceres is influenced by the draping and amplification of the IMF over its interior, which could result from either a mantle with moderate conductivity or an ocean with high conductivity. Grimm et al. (2021) show that EM sounding would present an ideal approach to detect a global ocean or even more shallow brine intrusions using either a transfer function approach with a combined lander-orbiter setup or an MT experiment.

In addition, Psyche is a metal-rich asteroid to be explored by a NASA mission of the same name arriving at the asteroid in 2029. When the mission was selected, the asteroid was expected to be entirely metal. This understanding has evolved and Psyche seems to be a mixed metal silicate world (Elkins-Tanton et al. 2020). It is unknown if the asteroid generates or has ever generated an internal magnetic field. Nevertheless, the mission includes a magnetometer as part of its payload to investigate this possibility (Weiss et al. 2023). While the distribution of metal within the body is unclear, electromagnetic sounding will be able to constrain the interior structure with 2 years of orbital data of the asteroid exposed to the solar wind (Weiss et al. 2023).

## Future Prospects for Electromagnetic Sounding in the Solar System

Magnetometry missions that are either in orbit or actively being prepared are shown in Fig. 1 and have been described in this review. Upcoming destinations include Mercury, the Moon, Mars, the asteroid Psyche, and the Jupiter system. Looking ahead, proposed missions to Enceladus and Uranus present opportunities to address significant knowledge gaps, allowing us to explore largely unknown worlds. Electromagnetic sounding, in particular, offers tools for investigating areas that are otherwise inaccessible. However, planning missions to the outer Solar System requires foresight and preparation that often span decades. This is why it is crucial for space agencies to actively prioritize and initiate planning now to ensure continuity in future exploration and discovery.

While EM sounding offers critical insights into planetary interiors, its full potential is realized when integrated with complementary datasets from other scientific disciplines. The importance of missions like InSight on Mars and Apollo on the Moon, which have collected complementary data on planetary interiors, cannot be overstated. For example, joint inversions of lunar seismic and EM data have yielded robust information on the internal structure of the Moon (Khan et al. 2014). For Mars, seismically derived interior structure models might be the key for future successful EM sounding. The value of multiparameter observations is further demonstrated by planned lunar magnetotelluric sounders, which will be collocated with heat flow probes to enhance interpretations of thermal and compositional properties (Fuqua Haviland et al. 2022). Geodetic measurements have also played a foundational role in constraining interior structure and, in some cases, have been key to identifying potential Ocean Worlds (Nimmo and Pappalardo 2016).

In summary, the future of EM sounding in planetary sciences is promising. As missions reach new destinations and as instrumentation continues to improve, we can anticipate groundbreaking discoveries about the hidden interiors of Solar System bodies. As such, the coming decades hold great potential for advancing our understanding of planetary evolution, habitability, and the diversity of worlds beyond Earth. I will conclude by expressing excitement on the data to come: The future looks bright.
